# Accurate Free Energies of Aqueous Electrolyte Solutions from Molecular Simulations with Non-polarizable Force Fields

**DOI:** 10.1021/acs.jpclett.4c00428

**Published:** 2024-04-18

**Authors:** Parsa Habibi, H. Mert Polat, Samuel Blazquez, Carlos Vega, Poulumi Dey, Thijs J. H. Vlugt, Othonas A. Moultos

**Affiliations:** † Engineering Thermodynamics, Process & Energy Department, Faculty of Mechanical Engineering, Delft University of Technology, Leeghwaterstraat 39, 2628 CB Delft, Netherlands; ‡ Department of Materials Science and Engineering, Faculty of Mechanical Engineering, Delft University of Technology, Mekelweg 2, 2628 CD Delft, Netherlands; § Departamento de Química Física, Facultad de Ciencias Químicas, Universidad Complutense de Madrid, 28040 Madrid, Spain

## Abstract

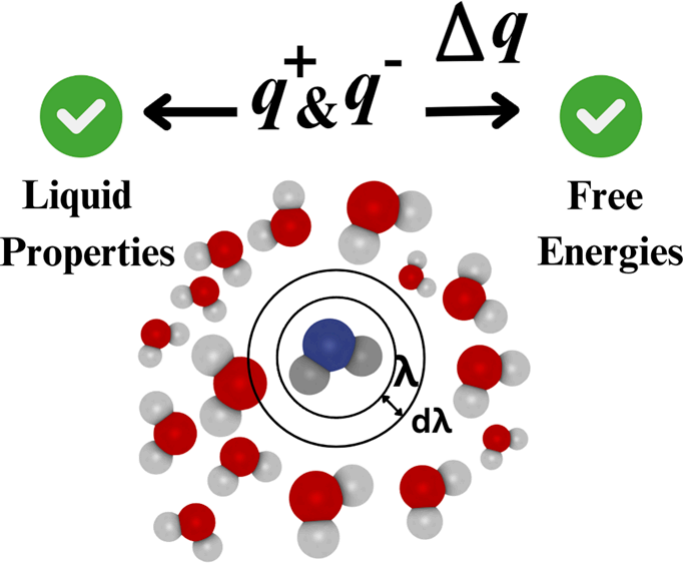

Non-polarizable force fields fail to accurately predict free energies of aqueous electrolytes without compromising the predictive ability for densities and transport properties. A new approach is presented in which (1) TIP4P/2005 water and scaled charge force fields are used to describe the interactions in the liquid phase and (2) an additional Effective Charge Surface (ECS) is used to compute free energies at zero additional computational expense. The ECS is obtained using a single temperature-independent charge scaling parameter per species. Thereby, the chemical potential of water and the free energies of hydration of various aqueous salts (e.g., NaCl and LiCl) are accurately described (deviations less than 5% from experiments), in sharp contrast to calculations where the ECS is omitted (deviations larger than 20%). This approach enables accurate predictions of free energies of aqueous electrolyte solutions using non-polarizable force fields, without compromising liquid-phase properties.

Accurate modeling of vapor–liquid equilibria (VLE) of aqueous electrolyte systems is crucial for a variety of applications, such as wastewater treatment,^1^ water electrolysis,^2−4^ and biomedical applications.^5,6^ Modeling aqueous electrolytes is a significant challenge as a result of long-range electrostatic interactions that make solutions highly non-ideal.^7,8^ Significant efforts are made to develop analytical models [equations of state (EOS)] for aqueous electrolytes.^9−13^ Although these models are computationally efficient, they rely on existing thermophysical data for parametrization and do not offer atomistic insight.^8,13^ Molecular simulation is a powerful tool for atomistic modeling and predicting thermodynamic and transport properties of aqueous electrolyte solutions at different temperatures, pressures, and electrolyte concentrations.^14−17^ The accuracy of molecular simulations depends upon the potential energy surface (PES) that is used to compute the interactions between different species.^18−21^ The PES of aqueous electrolyte solutions can be computed from *ab initio* calculations or semi-empirical force fields.^19,20,22^

For this purpose, many classical force fields for water have been developed.^23−29^ TIP4P/2005^23^ is a computationally efficient and popular water force field, which accurately predicts many properties of water, such as the shear viscosity, diffusivity, density, temperature of maximum density, and surface tension, despite being rigid and non-polarizable.^15,21,23,30^ Clearly, the effective interactions of TIP4P/2005 (dictated by the relative energy differences in the PES^18^) in the liquid phase are well-described.^18,30^ Despite this, the TIP4P/2005 force field does not yield accurate predictions of the VLE of water, because predictions for vaporization enthalpies and saturated vapor pressures are poor.^29,31,32^ Describing the VLE of water requires accurate modeling of (1) effective interactions between water molecules and (2) the excess chemical potential (with respect to the ideal gas reference state) of the liquid phase (μ_w_^ex^)^29^ (dictated by the absolute value of the PES^18^), because the coexistent pressures have an exponential dependency upon μ_w_^ex^.^33^ TIP4P/2005 consistently underestimates μ_w_^ex^ compared to experiments (e.g., by ca. 10% at 300 K), resulting in a significant underestimation of experimental saturated vapor pressures (by a factor of ca. 4 at 300 K).^29^ The second virial coefficients of TIP4P/2005 are also inaccurate compared to experimental data.^34,35^

Rigid non-polarizable water force fields that accurately capture experimental μ_w_^ex^ and the vaporization enthalpy of water, e.g., SPC,^24^ TIP4P,^23^ and TIP4P/μ^29,36^ (defined in the Supporting Information of ref (29)), poorly predict other important properties of the liquid phase (e.g., transport properties) compared to TIP4P/2005.^18,23,24,29,36^ It becomes clear that modeling both transport properties of water and μ_w_^ex^ is not possible using available non-polarizable force fields.^18,24^ Already in 1987, Berendsen et al.^24^ discussed this issue: to obtain effective interactions between water molecules in the liquid phase (thereby capturing experimental transport properties), the absolute value of the charges in the water force field needs to be enhanced to account for the polarization energy of water (i.e., “the missing term” in non-polarizable force fields mentioned in the title of the famous paper by Berendsen and co-workers^24^). Explicitly accounting for “the missing term” in non-polarizable force fields automatically results in an overestimation of the heat of vaporization and, hence, poor predictions of the VLE of water.^18,24,31^ Some polarizable force fields (e.g., BK3^25^ and HBP^26^) capture the VLE of water without compromising the transport properties of the liquid phase but at the cost of higher complexity, significantly higher computational time (usually by a factor of ca. 3–10),^26,31,37,38^ and lack of transferability.^25,26,38^ Therefore, non-polarizable force fields will likely remain popular for large-scale classical molecular simulations.

On the basis of TIP4P/2005 water, different force fields for salts (e.g., NaCl, KCl, and KOH) have been developed.^14,36,39−41^ The charges of ion force fields are commonly scaled down (usually by a factor of 0.85^40^ or 0.75^14,41^)^42,43^ to account for the effective charge screening that occurs in the aqueous medium.^40,42^ Charge scaling follows from the Electronic Continuum Correction and accounts for polarizability of ions in a mean-field way.^42,43^ Using the “scaled charge” force fields of Madrid-2019^40^ (scaled charges of +0.85/–0.85), Madrid-Transport (scaled charges of +0.75/–0.75),^41^ and the Delft Force Field of OH^–^ (DFF/OH^–^)^14^ (scaled charge of −0.75), many of the properties of aqueous NaCl, KCl, NaOH, and KOH solutions, such as densities, viscosities, and interfacial tensions, and their temperature dependence can be accurately computed.^14,16,17,40,41^ Force fields with integer charges of ions (e.g., +1/–1 for Na^+^/Cl^–^), such as the Joung–Cheatam force field,^44^ significantly overestimate the change in liquid-phase viscosities and ion diffusivities in concentrated solutions (i.e., close to the solubility limit) with respect to the pure solvents.^40^ The infinite dilution free energies of hydration of salts can be accurately captured using available integer charge force fields, whereas scaled charge force fields of ions deviate by ca. 20–30% compared to experiments.^45,46^

Recently, Han et al.^30^ have successfully simulated the dielectric constant of water using non-polarizable force fields. This study shows that the charges used in TIP4P/2005 water should only be used to model the PES, from which effective interactions between molecules are computed, and a different set of charges (derived from quantum mechanical simulations) should be used to model the dipole moment of the aqueous system, from which the dielectric constant is computed.^17,30^ Similarly, Blazquez et al.^17^ reproduced the experimental electrical conductivities of aqueous NaCl and KCl solutions up to the solubility limit (1) using non-polarizable scaled charge force fields to describe the PES of ions and (2) using integer charges to compute the dipole moment of the aqueous solution from which electrical conductivities are calculated.

Here, we introduce a new approach to accurately compute free energies of aqueous electrolyte solutions using non-polarizable force fields without compromising the predictive ability for transport and thermodynamic properties of the liquid phase. The PES is modeled using the TIP4P/2005^23^ force field and the Madrid-2019^40^ scaled charge ions, whereas a different set of charges, hereafter referred to as the effective charge surface (ECS), is used to compute excess chemical potentials in the liquid phase. The ECS corrects for the effect of both polarization energy (i.e., “the missing term” of Berendsen et al.^24^) and charge scaling^42,43^ on the computed free energies. We show that, using an ECS trained for TIP4P/2005^23^ water at 350 K, the experimental excess chemical potential of water along the liquid–vapor coexistence line can be reproduced within ca. 1% at a temperature range of 300–500 K, thereby yielding accurate predictions for the saturated vapor pressures. Similarly, a single-parameter ECS trained on the free energy of hydration of Madrid-2019 NaCl in water at 298 K corrects the free energies of hydration for the Madrid-2019 family of both mono- and divalent salts, such as LiCl, KCl, MgCl_2_, and CaCl_2_, with ca. 5% accuracy from the experimental data of Marcus.^47^ On the basis of the computed excess chemical potential of pure water using the ECS, we correct the excess chemical potentials of water/salt mixtures by applying a free energy correction to the partition function of the system. Using this, we compute liquid/vapor coexistence densities of the water/NaCl system at 350 K up to 6 mol of NaCl/kg of water. Our simulations show excellent agreement (within error bars) with experiments, in sharp contrast to simulations that do not have this correction (e.g., ca. a factor of 4 deviation for saturated vapor densities at 350 K).

The workflow of our method is shown in Figure 1. Continuous fractional component Monte Carlo (CFCMC)^48−50^ simulations in the isobaric–isothermal (*NPT*) ensemble are performed to simulate pure water and aqueous electrolyte solutions [i.e., NaCl(aq), KCl(aq), MgCl_2_(aq), and CaCl_2_(aq)] using the BRICK-CFCMC open source software.^51,52^ We define charge-neutral “fractional groups”, which contain one or more ions or molecules.^51^ For water, the fractional group contains a single molecule of water. For salts, it contains all ions in the molecule (e.g., for MgCl_2_, the fractional group consists of one Mg^2+^ and two Cl^–^ ions). These molecules or ions have their interactions modified by the order parameter λ. At λ = 0, the molecules/ions in the fractional group behave as ideal gas particles, and at λ = 1, the fractional group fully interacts with the surrounding. Sampling of the λ space is performed in CFCMC simulations. Details of CFCMC simulations can be found in the Supporting Information and in refs (48 and 51). We first simulate pure water in the absence of ions. With the exception of the single fractional molecule, all water molecules interact via the TIP4P/2005^23^ force field. The fractional H_2_O molecule is a new species (i.e., ECS-TIP4P/2005 with parameters listed in Table S1 of the Supporting Information) with the Lennard-Jones (LJ) parameters and geometry of TIP4P/2005 but with a different set of charges (i.e., *q*_ECS_^+^ for the charge on H atoms and *q*_ECS_^–^ for the charge on the M site, which are obtained by multiplying the charges of TIP4P/2005 by a factor of 0.965). This ensures that bulk properties of liquid water (i.e., densities and transport properties) are computed using the TIP4P/2005 water force field, while the ECS charges are used when sampling the λ space and calculating the excess chemical potential of pure water (μ_w,ECS,*m* = 0_^ex^; i.e., at the molality of the salt, *m* = 0 mol of salt/kg of water). To compute the excess chemical potential of water at finite salt concentrations, μ_w,ECS,*m* = 0_^ex^ is used to compute a free energy correction (ε_w_) for water
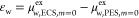
1where μ_w,PES,*m* = 0_^ex^ refers to the excess chemical potential of pure water computed using the PES charges (i.e., TIP4P/2005). ε_w_ depends only upon the temperature (*T*) because it is calculated along the liquid branch of the vapor–liquid coexistence curve (i.e., when evaluating eq 1, the pressure, *P*, should be fixed to the saturated vapor pressure). ε_w_ is applied as a background energy in the isolated molecule partition function of water and changes the partition function (*Q*_*NPT*_) of the system (here shown for the isobaric–isothermal ensemble)
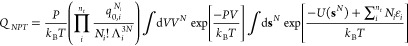
2where *V*, *N*, **s**^*N*^, *k*_B_, Λ_*i*_, and *n*_*t*_ refer to the system volume, total number of molecules, scaled coordinate vector of all molecules, Boltzmann constant, thermal wavelength of species *i*, and total number of species types, respectively. *q*_0,*i*_, *N*_*i*_, and ε_*i*_ refer to isolated molecular partition functions (excluding the translation part), the number of molecules of species *i*, and the free energy correction for species *i*, respectively. The derivation of eq 2 after applying the free energy correction ε_*i*_ to the isolated molecule partition function of species *i* is shown in the Supporting Information. ε_*i*_ is not a function of *V* and **s**^*N*^, and therefore, it does not influence the density, virial pressure, liquid structure, or transport properties at a given temperature or pressure. The chemical potential of species *i* (μ_*i*_) can be computed using
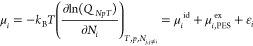
3where μ_*i*_^id^ is the ideal gas contribution and μ_*i*,PES_^ex^ is the excess chemical potential computed from the PES. The chemical potential, μ_*i*_, at *m* = 0 is shifted to equal the values obtained using the ECS. After the free energy correction is applied, the charges of TIP4P/2005^23^ can be used to compute excess chemical potentials at finite salt concentrations, because changes in the chemical potential as a function of *m* can be computed accurately using the PES.^39^ Activities (*a*_w_ = γ_w_*x*_w_, where γ_w_ and *x*_w_ refer to the activity coefficient and mole fraction of water, respectively) of water at different molalities of salts are computed using^53^
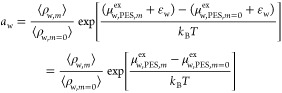
4where ⟨ρ_w,*m* = 0_⟩ and ⟨ρ_w,*m*_⟩ are the ensemble averaged number densities of water at molalities *m* and 0, respectively. μ_w,PES,*m*_^ex^ is the excess chemical potential of water computed using the PES at a molality *m*.^53^ ε_w_ does not depend upon *m* (at constant *T*), therefore, ε_w_ cancels out for all *m* (as shown in eq 4) when computing *a*_w_ (i.e., *a*_w_ only depends upon the PES).

**Figure 1:**
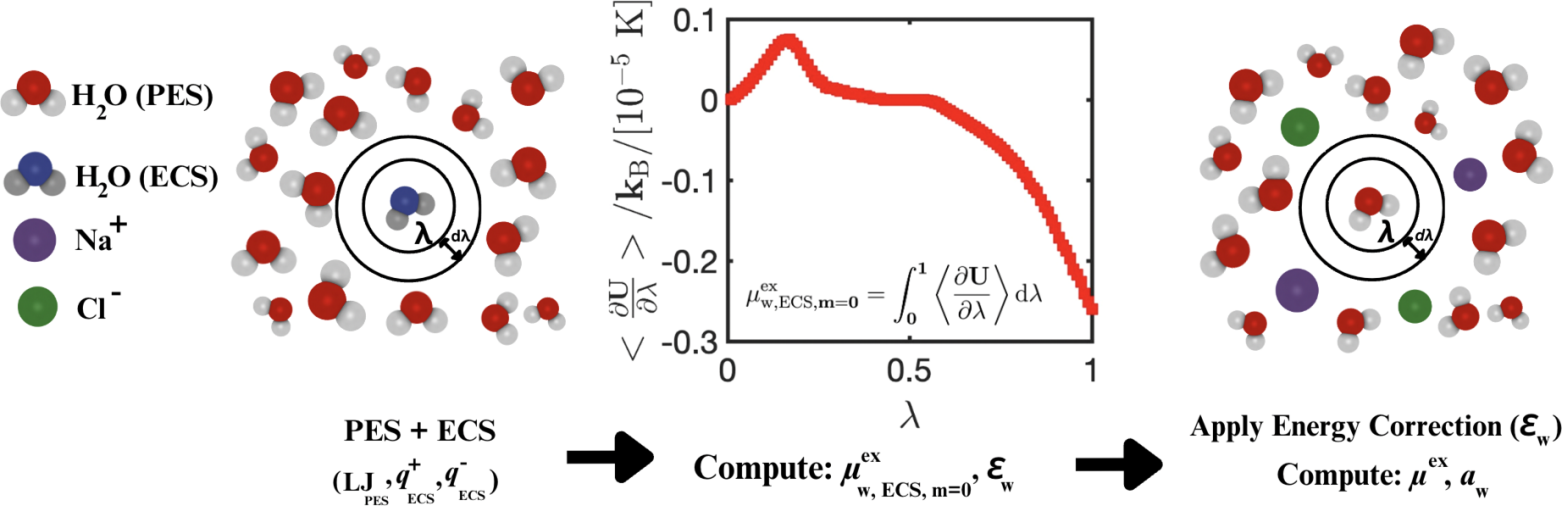
Schematic representation of the workflow used in this work.^48,49,51^ Excess chemical potentials (with respect to the ideal gas reference state) are computed using the BRICK-CFCMC software.^51,52^ The details for computing excess chemical potentials are discussed in the Supporting Information. The excess chemical potential of pure water (μ_w,ECS,*m* = 0_^ex^) at various temperatures (300–500 K) is computed using a single fractional molecule of water, with the same LJ parameters and geometry as TIP4P/2005 but with different temperature-independent charges (i.e., *q*_ECS_^+^ and *q*_ECS_^–^). The computed (μ_w,ECS,*m* = 0_^ex^) value is used to construct a temperature-dependent free energy correction (ε_w_) with which μ_w_^ex^ of water at different salt concentrations can be computed.

The ECS approach is used to correct the excess chemical potential of TIP4P/2005^23^ water. Panels (a) and (b) of Figure 2 show the computed liquid densities and excess chemical potentials of water at the simulated vapor–liquid coexistence line at 300–500 K for TIP4P/2005,^23^ TIP4P/μ,^29^ and the ECS approach of this work. As shown in panels (a) and (b) of Figure 2, TIP4P/2005^23^ accurately predicts the liquid densities at coexistence, but it overestimates the attractive electrostatic interactions of water, leading to lower excess chemical potentials (and higher vaporization enthalpies).^18^ The overestimation of attractive electrostatic interactions is required to obtain correct intermolecular interactions, because polarization of water can then be modeled in a mean-field way as described by Vega^18^ and Berendsen et al.^24^

**Figure 2:**
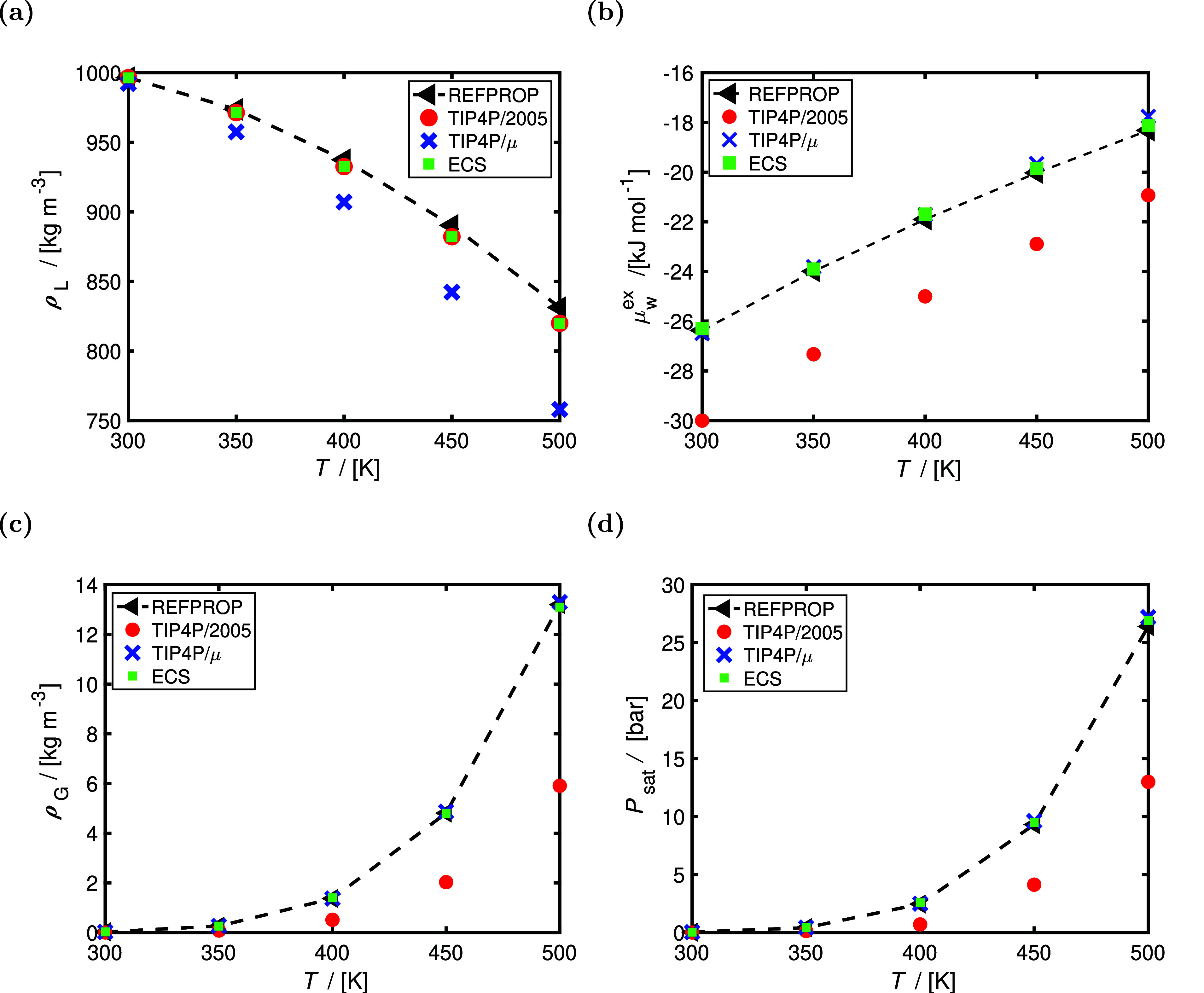
Computed (a) liquid densities (ρ_L_), (b) liquid-phase excess chemical potentials (μ_w_^ex^), and (c) gas densities (ρ_G_) as functions of temperature *T* along the vapor–liquid coexistence line of H_2_O. In panel (d), the saturated vapor pressure (*P*_sat_) of H_2_O on the vapor–liquid coexistence line is shown as a function of *T*. The computed results using the ECS developed in this work are compared to the results of the TIP4P/2005^23^ and TIP4P/μ^29^ water force fields and the experimental data obtained from REFPROP.^54,55^ For TIP4P/2005^23^ and TIP4P/μ, the results provided in ref (29) are used for the values of ρ_L_ and μ_w_^ex^.

At 300 K, the excess chemical potential of TIP4P/2005^23^ water in the liquid phase is −30.0 kJ/mol,^29^ while the experimental value is −26.37 kJ/mol (REFPROP, version 10,^54^ computed on the basis of IAPWS-95^55^). This underprediction of the excess chemical potentials for TIP4P/2005^23^ leads to a saturated vapor pressure that is ca. 4 times smaller than the experimental value at 300 K.^29,54,55^ Saturated vapor pressures (*P*_sat_) are related to the excess chemical potential of liquid water using^33,56^
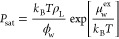
5where ϕ_w_ is the fugacity coefficient of water vapor at a given *T* and *P*_sat_. Equation 5 can be solved iteratively to obtain consistent *P*_sat_ and ϕ_w_. The derivation of eq 5 and the iterative scheme for a multicomponent mixture are discussed in section S2 of the Supporting Information. When *P*_sat_ is calculated using eq 5, it is assumed that the liquid phase is incompressible (i.e., ρ_L_ and μ_w_^ex^ computed in the liquid phase are not influenced by the pressure in the range of 1–50 bar). The TIP4P/2005 force field cannot accurately model the virial coefficient of water in the gas phase,^34,35^ and hence, it does not correctly describe deviations from the ideal gas behavior, leading to an inaccurate relation between *T*, *P*_sat_, and ϕ_w_. The relation between *T*, *P*_sat_, and ϕ_w_ is obtained from the Peng–Robinson EOS^57,58^ for water vapor, from which the saturated vapor densities (ρ_G_) follow for the ECS approach, TIP4P/2005,^23^ and TIP4P/μ. For TIP4P/2005^23^ and TIP4P/μ, the results provided in ref (29) are used for ρ_L_ and μ_w_^ex^.

The TIP4P/μ force field^29^ accurately describes the excess chemical potentials of liquid water (and thereby the saturated vapor pressures and densities), yet it underestimates the liquid densities at *T* > 300 K (as shown in Figure 2) because it cannot correctly capture the interactions between liquid water molecules. This also explains the ca. 30% lower viscosities for TIP4P/μ at 298 K and 1 bar compared to experiments.^36^ Using the ECS approach for TIP4P/2005, we accurately model the excess chemical potentials of pure liquid water compared to experiments for a wide temperature range (i.e., 300–500 K). The ECS charges are computed by multiplying the charges of TIP4P/2005^23^ by a temperature-independent charge scaling parameter equal to 0.965. This charge scaling parameter is obtained by fitting only the simulated excess chemical potential of liquid water at 350 K to the experimental value. As shown in Figure 2, the ECS approach leads to accurate modeling of the VLE of pure water without compromising liquid-phase densities and at the same computational expense as TIP4P/2005. The heat of vaporization computed using the ECS at 298 K and 1 bar is 45 ± 2 kJ mol^–1^ (obtained as explained in section S1 of the Supporting Information) and has a much closer agreement with experiments (44.01 kJ mol^–1^)^59^ than the TIP4P/2005 force field (i.e., 50.2 kJ mol^–1^).^59^

After obtaining the infinite dilution excess chemical potentials of water using the ECS, we computed the free energy correction (ε_w_) for TIP4P/2005 as a function of the temperature. ε_w_ is fitted as a linear function temperature using
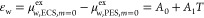
6where *A*_0_ (5.00 kJ mol^–1^) and *A*_1_ (−4.36 × 10^–3^ kJ mol^–1^ K^–1^) are the fitting parameters. Equation 6 provides an excellent fit for ε_w_ (within the error bars), as shown in Figure S2 of the Supporting Information.

Panels (a) and (b) of Figure 3 show the computed water activities and densities for TIP4P/2005^23^ combined with the Madrid-2019,^40^ Madrid-Transport,^41^ and the Joung–Cheatam^44^ NaCl force fields at 298 K and 1 bar. Activities of water at molality *m* (*a*_w_) are computed using eq 4 and do not depend upon ε_w_. As shown in Figure 3, activities of water are predicted best using scaled charge force fields, especially for concentrations higher than 4 mol of NaCl/kg of water, despite all force fields having accurate density predictions (within 1% agreement with the data of ref (61), as shown in Figure 3b). The Madrid-2019^40^ NaCl force field combined with TIP4P/2005^40^ predicts the activities of water with deviations smaller than ca. 3%. Accurate predictions of water activities indicate that the mean activity of the salt is correctly described as a result of the Gibbs–Duhem relation (binary mixture).^63^ Because the Madrid-2019^40^ NaCl and TIP4P/2005^23^ water combination has the best agreement with the experimental activities of water, these force fields are used with the ECS approach (i.e., using ε_w_) to simulate the coexistence vapor densities at 350 K at various NaCl molalities. The chemical potential of liquid water at molality *m* is equal to μ_w_(*m*) = μ_w_(*m* = 0) + *k*_B_*T* ln(*a*_w_), where μ_w_(*m* = 0) is the chemical potential at *m* = 0. In the ECS approach, the value of μ_w_(*m* = 0) is shifted by ε_w_ (eq 3), thereby changing *P*_sat_ (as computed by eq 5) and ρ_G_. As shown in panels (c) and (d) of Figure 3, the ECS approach results in perfect agreement (within the error bars) with the data of Clarke and Glew^62^ for the vapor-phase coexistence pressures and densities of water/NaCl mixtures, while TIP4P/2005^23^ at 350 K, underpredicts the vapor densities of water/NaCl by a factor of ca. 4. The saturated vapor pressures and densities of aqueous NaCl and CaCl_2_ solutions are computed at 300–350 K and shown in Figure S3 of the Supporting Information. As shown in this figure, the ECS approach combined with TIP4P/2005 and Madrid-2019 force fields can accurately capture the experimental saturated vapor pressures and densities for aqueous NaCl and CaCl_2_ solutions (within 5% deviation).

**Figure 3:**
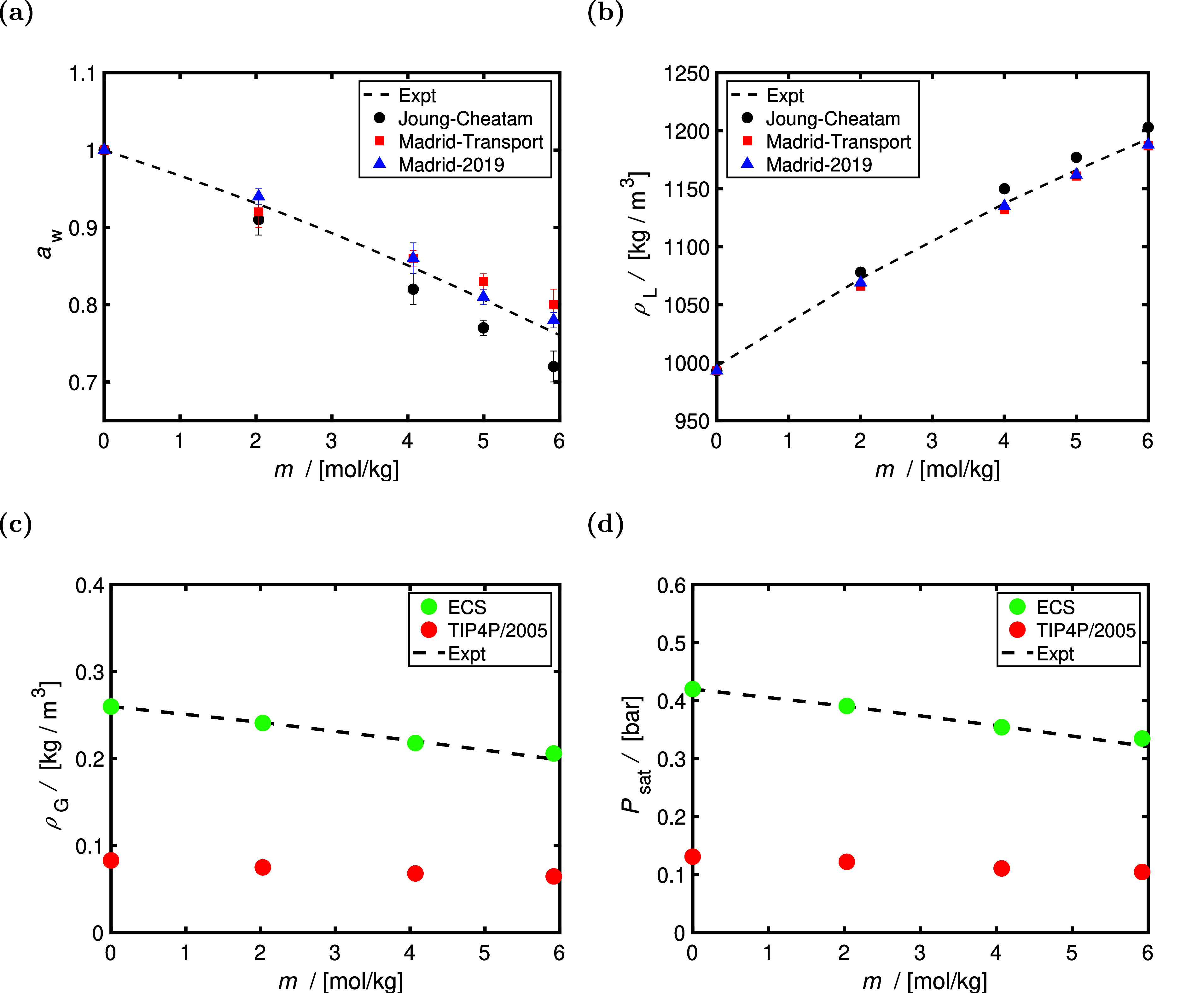
Computed (a) activities of water (*a*_w_) and (b) liquid densities (ρ_L_) at 298 K and 1 bar as a function of molality (*m*, in units of mol of NaCl/kg of water). The Madrid-2019,^40^ Madrid-Transport,^41^ and Joung–Cheatam^44^ NaCl force fields are combined with the TIP4P/2005^23^ water force field. The experimental correlation of Tang et al.^60^ is used for the activities of water at 298 K, and the experimental correlation of Laliberté and Cooper^61^ is used for the densities. Computed coexistence: (c) vapor densities (ρ_G_) and (d) saturated vapor pressures (*P*_sat_) of water at 350 K as a function of *m*. The liquid densities and excess chemical potentials used to calculate ρ_G_ and *P*_sat_ from eq 5 are computed at 1 bar. The ECS approach combined with TIP4P/2005^23^ water and Madrid-2019 NaCl force fields are used to compute ρ_G_, and the results are compared to the data of Clarke and Glew.^62^ The computed coexistence vapor densities of TIP4P/2005 and Madrid-2019 NaCl force fields without the ECS approach are shown for comparison in panel (c).

The computed free energies of hydration of several aqueous salts (i.e., NaCl, KCl, LiCl, MgCl_2_, and CaCl_2_) in TIP4P/2005 water are shown in Figure 4. The free energy of hydration of a salt refers to the free energy change associated with bringing an ion pair (ions infinitely apart) from a dilute gas phase to the aqueous phase.^64^ Even though scaled charge force fields can accurately predict experimental activities in aqueous electrolyte solutions (as shown in Figure 3a), the free energies of hydration computed using the scaled ion force fields of Madrid-2019^40^ (0.85 charge scaling for Na^+^, K^+^, Li^+^, Mg^2+^, Ca^2+^, and Cl^–^) underestimate the experimental values by ca. 20–30%. The Joung–Cheatam^44^ NaCl force field with integer charges of +1/–1 reproduces the free energies of hydration within 5% from experimental values;^44,46^ however, it largely overestimates the change in viscosities at higher molalities with respect to the pure solvent (i.e., at 298 K and 4 mol of NaCl/kg of water, the viscosity computed using the Joung–Cheatam NaCl force field combined with TIP4P/2005^23^ deviates by ca. 100% from experiments^40^). The ECS approach can be used to correct the free energies of hydration of these scaled charge ion force fields without influencing the predictive ability for the transport properties and activities of salt/water mixtures. For this, a single fractional group (i.e., consisting of a cation and an anion) molecule is introduced to a system with 300 TIP4P/2005^23^ water molecules. This fractional group uses the same LJ parameters as the Madrid-2019^40^ ion force fields but with different ion charges (i.e., +0.95/–0.95 for monovalent ions and +1.90/–1.90 for divalent ions). This ECS for the ion pair is trained at 298 K based on the free energy of hydration of an aqueous NaCl solution at infinite dilution using a single charge scaling parameter. For divalent ions, such as Mg^2+^, the ECS obtained for Na^+^ is multiplied by 2 (i.e., ion valency). Figure 4 clearly shows that the ECS approach leads to free hydration values that deviate by ca. 5% or less from the experimental data provided by Marcus^47^ for all ionic species considered. The free energies of hydration of Madrid-Transport^41^ ions (scaled charges of +0.75/–0.75) and DFF/OH^–^ (scaled charge of −0.75)^14^ can also be corrected using the ECS, as discussed in Figure S4 and Table S7 of the Supporting Information. This shows the applicability of the ECS approach to different ion force fields. It is important to note that these free energies of hydration of salts are computed at infinite dilution. To simulate the excess chemical potential of salts at finite concentrations, the free energy correction for the salt (ε_s_) needs to be computed using the same workflow followed for water (values of ε_s_ = μ_s,ECS,*m* = 0_^ex^ – μ_s,PES,*m* = 0_^ex^ at 298 K are listed in Tables S6 and S7 of the Supporting Information). This ensures that only the initial free energy offset is corrected, while energy differences (i.e., related to activities) are computed using the PES.

**Figure 4:**
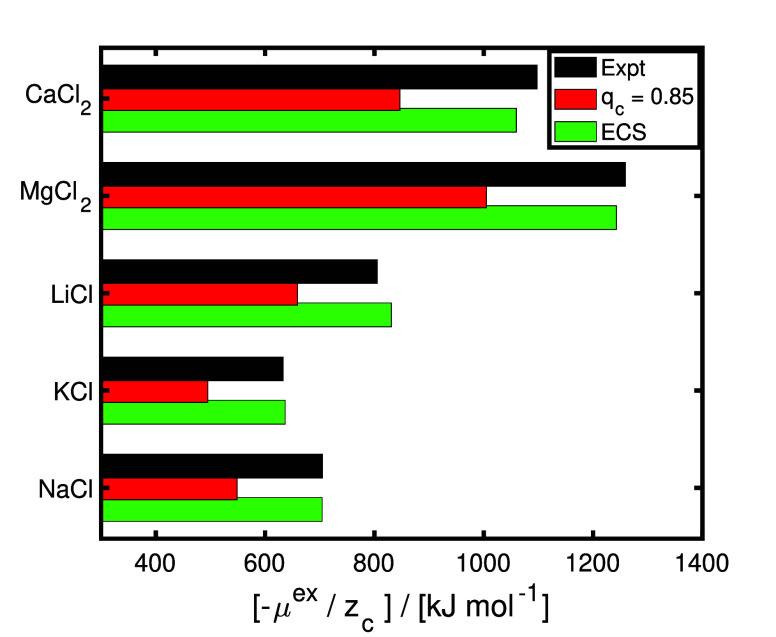
Computed infinite dilution excess chemical potentials (μ_ex_, i.e., free energies of hydration) at 298 K and 1 bar for aqueous NaCl, KCl, LiCl, MgCl_2_, and CaCl_2_ solutions at infinite dilution. μ_ex_ is normalized with respect to the integer cation charge (*z*_c_, i.e., 1 for Na^+^, K^+^, and Li^+^ and 2 for Ca^2+^ and Mg^2+^). The ion force fields of Madrid-2019^40^ (scaled charge of 0.85; *q*_c_ = 0.85 for Na^+^, K^+^, Li^+^, Mg^2+^, Ca^2+^, and Cl^–^) are considered. The TIP4P/2005^23^ water force field is used for all calculations. In the ECS approach, a single fractional group of cations and anions is used, with the same LJ parameters of Madrid-2019^40^ force fields. ECS charges of +0.95/–0.95 for monovalent ions and +1.90/–1.90 for divalent ions are used to sample the free energies of hydration (ECS charges are fitted only to the free energy of hydration of an aqueous NaCl solution at infinite dilution at 298 K and multiplied by the valency, i.e., 2, for divalent ions). The experimental data of Marcus^47^ are shown in black. All of the raw data are listed in Table S6 of the Supporting Information (along with the experimental free energy of hydration data of Marcus^47^ and Schmid et al.^65^).

In summary, we have shown that TIP4P/2005 can accurately model the vapor–liquid properties of aqueous electrolyte solutions, provided that an additional charge surface, the so-called ECS, is used to correct for the infinite dilution excess chemical potentials of water and salt. The excess chemical potential of water is corrected using the new ECS, and excellent agreement with experiments is obtained for both gas and liquid coexistence densities. A temperature-independent ECS trained at 350 K using a single charge scaling parameter (scaling factor of 0.965 with respect to the charges of TIP4P/2005^23^) can be used to model the infinite dilution excess chemical potentials of water from 300 to 500 K with differences smaller than 1% with respect to experiments. The excess chemical potentials of water at infinite dilution computed using the ECS are used to obtain a free energy correction that corrects the saturated vapor densities of water/NaCl systems. An ECS with charges of +0.95/–0.95 [e] for monovalent ions and +1.90/–1.90 [e] for divalent ions, trained only on an aqueous NaCl solution at infinite dilution (for divalent ions, the ECS is multiplied by the valency 2), successfully corrects the free energies of hydration of Madrid-2019^40^ force fields for aqueous KCl, LiCl, MgCl_2_, and CaCl_2_ solutions with deviations of ca. 5% or less from the experimental data of Marcus.^47^ The ECS approach enables accurate computation of free energies and VLE in large-scale molecular simulations using simple non-polarizable force fields, without compromising the predictive ability for thermodynamic and transport properties of the liquid phase. This method is transferable and can also be used for other non-polarizable water and ion force fields. An interesting future direction would be to investigate if the same approach can be used to accurately compute free energy differences between water (combined with salts) and ice, because these have a profound impact on computed nucleation rates of ice.

## Supporting Information Available

Force field parameters of water (Table S1), number of molecules used in CFCMC simulations (Table S2), vapor–liquid coexistence densities and excess chemical potentials of water at different temperatures (Table S3), liquid-phase densities and activities of aqueous NaCl solutions using different salt force fields (Table S4), liquid densities, excess chemical potentials (with respect to the ideal gas reference state) of water, and saturated vapor densities for aqueous NaCl and CaCl_2_ solutions at 300–350 K (Table S5), infinite dilution free energies of hydration for NaCl, KCl, LiCl, MgCl_2_, CaCl_2_, NaOH, and KOH in water (Tables S6 and S7), methodology details for the CFCMC simulations (section S1), derivation of the relation between the pressure and chemical potential and the iterative scheme to compute gas fugacities at phase coexistence (section S2), derivation of the partition function and the free energy correction for the liquid phase (section S3 and Figure S1), linear fit for the free energy corrections of water as a function of the temperature (Figure S2), saturated vapor densities of water for aqueous NaCl and CaCl_2_ solutions at 300 and 350 K (Figure S3), and infinite dilution free energies of hydration for NaCl, KCl, NaOH, and KOH in water (Figure S4) (PDF)
Transparent Peer Review report available (PDF)
