# Supplementary material for: Accurate Free Energies of Aqueous Electrolyte Solutions from Molecular Simulations with Non-polarizable Force Fields
Source: J Phys Chem Lett. 2024 Apr 18;15(16):4477–85. doi: 10.1021/acs.jpclett.4c00428 (PMC11057036; doi:10.1021/acs.jpclett.4c00428)
Supplement: Supplementary file 1 — jz4c00428_si_001.pdf [file jz4c00428_si_001.pdf]

Supporting Information: Accurate Free Energies of Aqueous Electrolyte Solutions from Molecular Simulations with Non-Polarizable Force Fields

Parsa Habibi,[†,‡] H. Mert Polat,[†] Samuel Blazquez,[¶] Carlos Vega,[¶] Poulumi Dey,[‡] Thijs J. H. Vlugt,[†] and Othonas A. Moultos[*,†]

†*Engineering Thermodynamics, Process & Energy Department, Faculty of Mechanical Engineering, Delft University of Technology, Leeghwaterstraat 39, 2628 CB Delft, The Netherlands*

‡*Department of Materials Science and Engineering, Faculty of Mechanical Engineering, Delft University of Technology, Mekelweg 2, 2628 CD Delft, The Netherlands*

¶*Depto. Química Física, Fac. Ciencias Químicas, Universidad Complutense de Madrid, 28040 Madrid, Spain*

E-mail: o.moultos@tudelft.nl

Force field parameters of water (Table S1); Number of molecules used in CFCMC simulations (Table S2); Vapor-Liquid coexistence densities and excess chemical potentials of water at different temperatures (Table S3); Liquid phase densities and activities of aqueous NaCl solutions using different salt force fields (Table S4); Liquid densities, excess chemical potentials (with respect to ideal gas reference state) of water, and the saturated vapor densities for aqueous NaCl and $CaCl_2$ solutions at 300-350 K (Table S5); Infinite dilution free energies of hydration for NaCl, KCl, LiCl, $MgCl_2$, $CaCl_2$, NaOH, and KOH in water (Tables S6-S7); Methodology details for the CFCMC simulations (section S1); Derivation of the relation between pressure and chemical potential and the iterative scheme to compute gas fugacities at phase coexistence (section S2); Derivation of the partition function and the free energy correction for the liquid phase (section S3 and Figure S1); Linear fit for the free energy corrections of water as a function of temperature (Figure S2); Saturated vapor densities of water for aqueous NaCl and $CaCl_2$ solutions at 300 K and 350 K (Figure S3); Infinite dilution free energies of hydration for NaCl, KCl, NaOH, and KOH in water (Figure S4).

Table S1: Parameters for TIP4P/2005,[1] ECS-TIP4P/2005 (note: here only used as a fractional molecule[2]), and TIP4P/μ (developed in the Supporting Information of Ref.[3]) for water. σ and ϵ are the Lennard-Jones parameters, q are atomic partial charges, and l is the bond length. σ and l are in units of Å, ϵ is in units of kJ/mol, and q is in units of the elementary charge e. In all force fields, the charge on O is on a massless site M (equidistant from both H atoms).

	TIP4P/2005[1]	TIP4P/μ[3]	ECS-TIP4P/2005
$\mathrm{H}-\widehat{\mathrm{O}}-\mathrm{H}\,(^{o})$	104.52	104.52	104.52
$l_{\mathrm{O-H}}$	0.9572	0.9572	0.9572
$l_{\mathrm{O-M}}$	0.1546	0.1546	0.1546
σ_{OO}	3.1589	3.1589	3.1589
σ_{HH}	0	0	0
ϵ_{OO}	0.774908	0.663989	0.774908
ϵ_{HH}	0	0	0
q_{O}	0	0	0
q_{M}	-1.1128	-1.06272	-1.073852
q_{H}	0.5564	0.53136	0.536926

Table S2: The numbers of water molecules or ions (N) used in Continuous Fractional Component Monte Carlo (CFCMC)[2,4–7] simulations to compute free energies of hydration of salts in water, excess chemical potentials (with respect to the ideal gas reference state), and activities of water. To compute the free energies of hydration of LiCl, NaCl, KCl, $MgCl_2$, and $CaCl_2$ at infinite dilution, 300 H_2O molecules (with no additional ion molecules) are used with a single fractional group for the salt molecule. Only for aqueous NaCl solutions, finite molalities (m in units of mol NaCl/kg water) are considered. The average box volume ($\langle V \rangle$) in units of Å^3 is shown for each molality at 298 K and 1 bar.

m	$N_{\mathrm{H_2O}}$	$N_{\mathrm{Na^+}}$	$N_{\mathrm{Cl^-}}$	$\langle V \rangle$
0	300	0	0	9041
0.93	300	5	5	9293
2.96	300	16	16	9874
5.00	300	27	27	10481

Table S3: Vapor-liquid coexistence densities for the liquid (ρ_L) and the gas (ρ_G) phases of water at different temperatures (T) for TIP4P/2005,[1] TIP4Pμ (developed in the Supporting Information of Ref.[3] and shown in Table S1), the ECS approach of this work, and REFPROP.[3,8,9] The liquid phase excess chemical potentials ($\mu_\mathrm{w}^\mathrm{ex}$) (with respect to the ideal gas reference state) in units of kJ mol^{-1} and the saturated vapor pressures (P_sat) in units of bar, are also listed. For TIP4P/2005[1] and TIP4P/μ, the results provided in Ref.[3] are used for ρ_L and $\mu_\mathrm{w}^\mathrm{ex}$. The ECS results are obtained using our approach in which the Potential Energy Surface of TIP4P/2005[1] is used to obtain ρ_L at a given T, and a single fractional molecule of water with the LJ parameters of TIP4P/2005 but with charges of TIP4P/2005 scaled by a factor 0.965 (as discussed in the main text) is used to compute $\mu_\mathrm{ex,L}$. P_sat of TIP4P/2005,[1] TIP4P/μ, and the ECS approach are computed using Eq. 5 of the main text and solved iteratively using the scheme discussed in section S2 of the Supporting Information. As explained in section S2, ρ_G for TIP4P/2005, TIP4P/μ, and the ECS approach at a given T and P_sat are obtained based on the Peng-Robinson equation of state for water vapor.[10,11] The values of REFPROP[8,9] for ρ_L, ρ_G, $\mu_\mathrm{ex,L}$, and P_sat at various T are also shown for comparison.

T / [K]	ρ_L / [kg m^{-3}]	ρ_G / [kg m^{-3}]	P_sat / [bar]	$\mu_\mathrm{ex,L}$ / [kJ mol^{-1}]
**TIP4P/2005**[1]				
300	996	0.0059	0.0082	-30.02
350	971	0.083	0.131	-27.33
400	933	0.514	0.94	-25.00
450	882	2.03	4.12	-22.89
500	820	5.91	13.0	-20.93
**TIP4P/μ**[3]				
300	992	0.024	0.0336	-26.49
350	958	0.270	0.435	-23.81
400	907	1.37	2.49	-21.71
450	842	4.87	9.61	-19.66
500	758	13.3	27.2	-17.76
**ECS-TIP4P/2005**				
300	996	0.026	0.036	-26.3
350	971	0.261	0.42	-23.9
400	933	1.40	2.58	-21.69
450	882	4.83	9.51	-19.86
500	820	13.1	26.9	-18.12
**REFPROP**[3,8,9]				
300	997	0.026	0.035	-26.37
350	974	0.26	0.42	-23.99
400	938	1.37	2.46	-21.90
450	890	4.81	9.32	-20.03
500	831	13.2	26.4	-18.32

Table S4: Computed liquid densities (ρ_L) in units of kg m^{-3} and activities of water ($a_w = \gamma_w x_w$, where γ_w and x_w refer to the activity coefficient and mole fraction of water, respectively) for aqueous NaCl solutions at different salt molalities (m, in units of mol NaCl / kg water) at 298 K and 1 bar. The TIP4P/2005[1] water force field is combined with three different NaCl force fields, i.e., Madrid-2019,[12] Madrid-Transport,[13] and the Joung-Cheatam[14] force fields. For the combination of TIP4P/2005[1] water and Joung-Cheatam[14] NaCl, the force field parameters are listed in Ref.[15] σ_{a_w} is the standard deviation of a_w.

NaCl Model	m / [mol salt/kg water]	ρ_L / [kg m^{-3}]	a_w	σ_{a_w}
Madrid-2019[12]	2.03	1069	0.94	0.01
	4.07	1135	0.86	0.02
	4.99	1162	0.81	0.01
	5.92	1188	0.78	0.01
Madrid-Transport[13]	2.03	1066	0.92	0.02
	4.07	1132	0.86	0.02
	4.99	1162	0.81	0.01
	5.92	1187	0.8	0.02
Joung-Cheatam[14]	2.03	1078	0.91	0.02
	4.07	1150	0.82	0.02
	4.99	1177	0.77	0.01
	5.92	1203	0.72	0.02

Table S5: Computed liquid densities (ρ_L) in units of kg m^{-3}, excess chemical potentials of liquid water ($\mu_{ex,L}$, i.e., with respect to ideal gas reference state) in units of kJ mol^{-1}, the saturated vapor densities of water (ρ_G) in units of kg m^{-3}, and saturated vapor pressures (P_{sat}) in units of bar at different temperatures (T in units of K). The ECS approach (as described in the main text) is combined with the TIP4P/2005[1] and the Madrid-2019[12] force fields of NaCl and $CaCl_2$. The computed results without the use of the ECS are also shown for comparison. σ_{ρ_L}, $\sigma_{\mu_{ex,L}}$, σ_{ρ_G}, and $\sigma_{P_{sat}}$ are the standard deviations of ρ_L, $\mu_{ex,L}$, ρ_G, and P_{sat}, respectively (in the same units). Note that $\mu_{ex,L}$ for TIP4P/2005 and ECS + TIP4P/2005 are computed using the same simulation, with the exception that a temperature-dependant free energy correction (as explained in the main text) is applied to the ECS results. This leads to the same $\sigma_{\mu_{ex,L}}$ values at different salt molalities. ρ_L and $\mu_{ex,L}$ used to calculate ρ_G and P_{sat} from Eq. 5 of the main text are computed at 1 bar.

T	m	ρ_L	σ_{ρ_L}	$\mu_{ex,L}$	$\sigma_{\mu_{ex,L}}$	ρ_G	σ_{ρ_G}	P_{sat}	$\sigma_{P_{sat}}$
**ECS + TIP4P/2005 + Madrid-2019 NaCl**									
300	2.04	1068	1	-26.5	0.2	0.0229	0.0014	0.0317	0.0019
300	4.07	1134	1	-26.6	0.1	0.0216	0.0009	0.0300	0.0013
300	5.92	1186	1	-26.7	0.2	0.0200	0.0019	0.0277	0.0026
350	2.04	1039	1	-24.0	0.1	0.2428	0.0057	0.3910	0.0091
350	4.07	1101	1	-24.2	0.1	0.2200	0.0047	0.3543	0.0076
350	5.92	1150	1	-24.2	0.1	0.2078	0.0070	0.3348	0.0112
**TIP4P/2005 + Madrid-2019 NaCl**									
300	2.04	1068	1	-30.2	0.2	0.0052	0.0003	0.0072	0.0004
300	4.07	1134	1	-30.3	0.1	0.0049	0.0002	0.0068	0.0003
300	5.92	1186	1	-30.4	0.2	0.0045	0.0004	0.0063	0.0006
350	2.04	1039	1	-27.4	0.1	0.0756	0.0018	0.1221	0.0028
350	4.07	1101	1	-27.6	0.1	0.0686	0.0015	0.1107	0.0024
350	5.92	1150	1	-27.6	0.1	0.0648	0.0022	0.1046	0.0035
**ECS + TIP4P/2005 + Madrid-2019 $CaCl_2$**									
300	2.04	1153	2	-26.6	0.1	0.0217	0.0008	0.0301	0.0011
300	4.07	1273	3	-27.3	0.2	0.0154	0.0010	0.0213	0.0014
300	5.92	1348	5	-27.9	0.2	0.0115	0.0007	0.0159	0.0009
350	2.04	1125	1	-24.2	0.1	0.2283	0.0060	0.3677	0.0096
350	4.07	1245	2	-24.9	0.1	0.1650	0.0032	0.2660	0.0051
350	5.92	1326	2	-25.9	0.1	0.1091	0.0034	0.1761	0.0055
**TIP4P/2005 + Madrid-2019 $CaCl_2$**									
300	2.04	1153	2	-30.3	0.1	0.0049	0.0002	0.0068	0.0003
300	4.07	1273	3	-31.0	0.2	0.0035	0.0002	0.0048	0.0003
300	5.92	1348	5	-31.6	0.2	0.0026	0.0002	0.0036	0.0002
350	2.04	1125	1	-27.6	0.1	0.0699	0.0018	0.1128	0.0030
350	4.07	1245	2	-28.3	0.1	0.0506	0.0010	0.0817	0.0016
350	5.92	1326	2	-29.3	0.1	0.0335	0.0010	0.0541	0.0017

Table S6: Computed infinite dilution excess chemical potentials (i.e., free energies of hydration, with respect to the ideal gas reference state) in units of kJ mol^{-1} for the Madrid-2019[12] force field (scaled charges of +0.85/-0.85) and the ECS approach (as described in the main text) for aqueous NaCl, KCl, LiCl, $MgCl_2$, and $CaCl_2$ solutions at 298 K and 1 bar. For the ECS approach, the infinite dilution excess chemical potentials ($\mu^{ex}_{s,ECS,m=0}$) are computed using a fractional group containing the anion(s) and the cation with the same LJ parameters as the Madrid-2019[12] force field, but with different charges (+0.95/-0.95 for monovalent ions, and +1.90/-1.90 for divalent ions) corresponding to the ECS. This ECS is only trained based on $\mu^{ex}_{s,ECS,m=0}$ of aqueous NaCl (Madrid-2019[12]). In case of divalent ions (i.e., Mg^{2+} and Ca^{2+}), the value of the ECS charge obtained for Na^+ is multiplied by 2. $\sigma_{\mu^{ex}}$ is the standard deviation of the excess chemical potential and is in the same units. The free energy correction of each salt in the Madrid-2019[12] force fields (ϵ_s in units of kJ mol^{-1}) at 298 K (as defined in the main text) is shown. The experimental free energies of hydration of Marcus[16] and Schmid et al.[17] (in units of kJ mol^{-1}) are listed for comparison.

Salt	Madrid-2019[12]		ECS		ϵ_s	Exp.	Exp.
	$\mu^{ex}_{s,PES,m=0}$	$\sigma_{\mu^{ex}}$	$\mu^{ex}_{s,ECS,m=0}$	$\sigma_{\mu^{ex}}$		Marcus[16]	Schmid et al.[17]
NaCl	-548	2	-704.3	0.5	-156	-705	-744
KCl	-494	0.5	-636.7	0.6	-143	-635	-671
LiCl	-659.3	0.7	-831.4	0.7	-172	-815	-849
$MgCl_2$	-2009	1	-2485	2	-476	-2518	-
$CaCl_2$	-1691	1	-2119	1	-428	-2195	-

Table S7: Computed infinite dilution excess chemical potentials (i.e., free energies of hydration, with respect to the ideal gas reference state) in units of kJ mol^{-1} for the Madrid-Transport (MT) force field[13] (scaled charges of +0.75/-0.75), the Delft Force Field of OH^- (DFF/OH^-) (scaled charge of -0.75), and the ECS approach (as described in the main text) for aqueous NaCl, KCl, NaOH, and KOH solutions at 298 K and 1 bar. For the ECS approach, the infinite dilution excess chemical potentials ($\mu^{\text{ex}}_{\text{s,ECS},m=0}$) are computed using a fractional group containing the anion and the cation with the same LJ parameters as the MT force field[13] and DFF/OH^-, but with different charges (+0.90/-0.90 for monovalent ions) corresponding to the ECS. This ECS is only trained based on $\mu^{\text{ex}}_{\text{s,ECS},m=0}$ of aqueous NaCl (MT[13]). $\sigma_{\mu^{\text{ex}}}$ is the standard deviation of the excess chemical potential and is in the same units. The free energy correction of each salt in the Madrid-Transport[13] and DFF/OH^- force fields (ϵ_{s} in units of kJ mol^{-1}) at 298 K (as defined in the main text) is shown. The experimental free energies of hydration of Marcus[16] and Schmid et al.[17] (in units of kJ/mol) are listed for comparison.

Salt	MT[13] and DFF/OH^-[18]		ECS		ϵ_{s}	Exp.	Exp.
	$\mu^{\text{ex}}_{\text{s,PES},m=0}$	$\sigma_{\mu^{\text{ex}}}$	$\mu^{\text{ex}}_{\text{s,ECS},m=0}$	$\sigma_{\mu^{\text{ex}}}$		Marcus[16]	Schmid et al.[17]
NaCl	-470.8	0.9	-702.9	0.6	-232	-705	-744
KCl	-394.7	0.9	-596.6	0.6	-202	-635	-671
NaOH	-561.3	0.6	-826.6	0.6	-265	-795	-870
KOH	-492.4	1	-732	1	-240	-725	-797

S1 Methods

CFCMC simulations[2,4,6] are carried out using the open-source BRICK-CFCMC software package.[6,7] The simulation boxes are cubic and periodic boundary conditions are applied in all directions. The simulation boxes consist of 300 water molecules. The water force fields details and the number of salt molecules used in the simulations are provided in Tables S1 and S2 of the Supporting Information. The parameters for the Madrid-2019 force fields for Na^{+}, K^{+}, Li^{+}, Mg^{2+}, Ca^{2+}, and Cl^{-} can be found in Ref.[12] The parameters for the Madrid-Transport force fields for Na^{+}, K^{+}, and Cl^{-} and the Delft Force Field of OH^{-} can found in Refs.[13,18] The Ewald summation with a relative precision of 10^{-6} is used for long range electrostatic interactions.[19] A cutoff radius of 10 Å is used for the LJ interactions and the real space contribution of the Ewald-summation. Analytic tail corrections for the LJ interactions are applied for computing energies and pressures. To compute excess chemical potentials (with respect to the ideal gas reference state), charge-neutral "fractional groups" are defined, which contain one or more molecules or ions.[6] For water, the fractional group contains a single molecule of water. For salts, it contains all ions in the salt molecule (e.g., for $MgCl_2$ the fractional group consists of one Mg^{2+} and two Cl^{-} ions). These molecules or ions in the fractional group have their interactions scaled by an order parameter λ. At $\lambda = 0$, the species in the fractional group behaves as an ideal gas particles, and at $\lambda = 1$ the fractional group fully interacts with the surroundings. More details on the implementation of fractional molecules in BRICK-CFCMC can be found in Refs.[6,7] The excess chemical potentials of water ($\mu_{\mathrm{w}}^{\mathrm{ex}}$) and aqueous salts (i.e., NaCl, KCl, LiCl, $MgCl_2$, $CaCl_2$, NaOH, and KOH) are computed using two different methods as outline below.

S1.1 μ^{ex} and vaporization enthalpy of water

The Wang-Landau algorithm[20,21] is used to construct a biasing weight function for λ ($W(\lambda)$) to overcome energy barriers in the λ-space and ensure a flat observed probability distribution

of λ ($p_{\mathrm{o}}(\lambda)$).[18,22] 100 bins are used to create a histogram with which the observed probability of occurrence of λ is computed. The Boltzmann averaged probability distributions ($p(\lambda)$) can be computed from $W(\lambda)$ and $p_{\mathrm{o}}(\lambda)$ using:[18]

$$p(\lambda) = \frac{\langle p_{\mathrm{o}}(\lambda) \exp\left[-W(\lambda)\right]\rangle}{\langle \exp\left[-W(\lambda)\right]\rangle} \tag{S1}$$

$\mu_{\mathrm{w}}^{\mathrm{ex}}$ is computed from $p(\lambda)$ using:[18]

$$\mu_{\mathrm{w}}^{\mathrm{ex}} = -k_{\mathrm{B}}T \exp\left[\frac{p(\lambda=1)}{p(\lambda=0)}\right] \tag{S2}$$

where k_{B}, T, $p(\lambda = 1)$, and $p(\lambda = 0)$ refer to the Boltzmann constant, absolute temperature, Boltzmann averaged probability distribution at $\lambda = 1$ and $\lambda = 0$, respectively. For all simulations for computing $\mu_{\mathrm{w}}^{\mathrm{ex}}$, 5×10^5 equilibration cycles are carried out followed by 1.5×10^6 production cycles. Each cycle contains N number of trial moves, with N corresponding to the total number of molecules (with a minimum of 20). Trial moves are selected with the following probabilities: 1% volume changes, 35% translations, 29% rotations, 25% λ changes, and 10% reinsertions of the fractional molecules at random locations inside the simulation box. The maximum displacements for volume changes, molecule translations, rotations, and λ changes are adjusted to obtain ca. 50% acceptance of trial moves. 100 independent simulations are performed. $p(\lambda)$ is averaged from 20 simulations, resulting to 5 independent averaged distributions. From these 5 averaged probability distributions, the excess chemical potentials of water are calculated to obtain a mean value and a standard deviation. All the raw data for the excess chemical potentials of water are shown in Tables S3 and S5 of the Supporting Information.

The vaporization enthalpy of ECS-TIP4P/2005 water (see Table S1 for the force field parameters) at 298 K and 1 bar is shown in the main text and is computed using:[1]

$$\Delta H_{\mathrm{vap}} = \Delta H_{\mathrm{G}} - \Delta H_{\mathrm{L}} \tag{S3}$$

where ΔH_{vap} is the vaporization enthalpy of ECS-TIP4P/2005 water. ΔH_{G} and ΔH_{L} are the enthalpy changes in the gas and liquid phase after adding an ECS-TIP4P/2005 water molecule. ΔH_{L} is computed by subtracting the average enthalpy of a system containing 300 TIP4P/2005 water molecules plus an additional molecule of ECS-TIP4P/2005 from the average enthalpy of a system containing only 300 TIP4P/2005 water molecules at a constant pressure of 1 bar. 100 independent MC simulations are carried out and the enthalpies are averaged from 20 simulations, resulting to 5 independent averaged ΔH_{L} values. 5×10^5 equilibration cycles are carried out followed by 1×10^6 production cycles. Trial moves are selected with the following probabilities: 1% volume changes, 50% translations, and 49% rotations. Assuming an ideal gas (as the fugacity coefficient of water at 298 K at the saturation pressure is statistically not different from unity[8,9]), ΔH_{G} is approximated as $4k_BT$ (i.e., the kinetic energy contribution plus the PV contribution for an ideal gas, where P and V are the system pressure and volume, respectively).[1]

S1.2 μ^{ex} of aqueous salts

The excess chemical potentials of aqueous salts ($\mu_{\text{s}}^{\text{ex}}$) are computed using thermodynamic integration,[7] as the free energy barriers in the λ-space are too large (exceeding 100 k_BT) to construct biasing weight functions using the Wang-Landau algorithm.[7] $\mu_{\text{s}}^{\text{ex}}$ is computed using thermodynamic integration:

$$\mu_{\text{s}}^{\text{ex}} = \int_0^1 \text{d}\lambda \left\langle \frac{\partial U}{\partial \lambda} \right\rangle_P \tag{S4}$$

where $\langle \frac{\partial U}{\partial \lambda} \rangle$ is the average partial derivative of the internal energy of the system with respect to λ.[7] Thermodynamic integration is performed using a path at constant pressure (P). 100 different simulations are carried at fixed values of λ ranging from 0 to 1. For each simulation, 5×10^5 equilibration cycles are carried out, followed by 1×10^6 production cycles. Trial moves are selected with the following probabilities: 1% volume changes, 50% translations,

and 49% rotations. The maximum displacements for volume changes, molecule translations, and rotations are adjusted to obtain ca. 50% acceptance of trial moves. For each simulation, $\langle \frac{\partial U}{\partial \lambda} \rangle$ is averaged over 0.2×10^6 cycles, thereby giving 5 independent values for $\langle \frac{\partial U}{\partial \lambda} \rangle$ values for 1×10^6 production cycles. 5 different values of $\mu_{\mathrm{s}}^{\mathrm{ex}}$ are obtained (from Eq. S4) with which the mean and standard deviations of $\mu_{\mathrm{s}}^{\mathrm{ex}}$ are computed. All the raw data for the MC simulations of aqueous salts are shown in Tables S4 and S5 of the Supporting Information.

S2 Derivation of the Relation Between Pressure and Chemical Potential and the iterative scheme to compute gas fugacities

We derive an equation relating the pressures and composition of a multi-component gas mixture to the excess chemical potentials in the liquid phase (Eq. 5 of the main text). At a given temperature (T) and pressure (P), chemical equilibrium dictates that chemical potentials of each species in the gas phase are equal to the chemical potential of the same species in the liquid phase:[23,24]

$$k_{\mathrm{B}}T\ln\left(\frac{\rho_{\mathrm{G},i}}{\rho_0}\right)+\mu^{\mathrm{ex}}_{\mathrm{G},i}=k_{\mathrm{B}}T\ln\left(\frac{\rho_{\mathrm{L},i}}{\rho_0}\right)+\mu^{\mathrm{ex}}_{\mathrm{L},i} \tag{S5}$$

where k_{B}, $\rho_{\mathrm{G},i}$, $\rho_{\mathrm{L},i}$, $\mu^{\mathrm{ex}}_{\mathrm{G},i}$, and $\mu^{\mathrm{ex}}_{\mathrm{L},i}$ refer to the Boltzmann constant, gas phase number density of species i, liquid phase number density of species i, excess chemical potential (with respect to the ideal gas reference state) in the gas phase for species i, and the excess chemical potential in the liquid phase for species i, respectively. ρ_0 is a reference number density (equal to 1 m^{-3}) to make the arguments of the natural logarithm unitless. $\mu^{\mathrm{ex}}_{\mathrm{G},i}$ is related to the fugacity coefficient of species i (ϕ_i) in the gas phase using:[24]

$$\mu^{\mathrm{ex}}_{\mathrm{G},i}=k_{\mathrm{B}}T\ln\left(\frac{y_iP\phi_i}{k_{\mathrm{B}}T\rho_{\mathrm{G},i}}\right) \tag{S6}$$

where y_i is the mole fraction of species i in the gas phase. This equation is derived in the supporting information of Ref.[24] After substituting $\mu^{\mathrm{ex}}_{\mathrm{G},i}$ in Eq. S5 by Eq. S6, we obtain:

$$y_iP=\frac{k_{\mathrm{B}}T\rho_{\mathrm{L},i}}{\phi_i}\exp\left[\frac{\mu^{\mathrm{ex}}_{\mathrm{L},i}}{k_{\mathrm{B}}T}\right] \tag{S7}$$

For a multi-component mixture with n_t number of species, we have n_t+1 number of unknowns (i.e., P and n_t values for y_i) and n_t+1 number of equations (i.e., n_t different versions of

Eq. S7 for each species, and $\sum_i^{n_t} y_i = 1$), which we can solve iteratively using an equation of state for the gas phase. In this work, we only have a single species (water) in the gas phase (i.e., $y_\mathrm{w} = 1$). Eq. S7 is solved iteratively by initially starting with $\phi_\mathrm{w} = 1$. After computing a first estimate of P using Eq. S7, an improved estimate for ϕ_w is computed using the Peng-Robinson equation of state.[10,11] This iterative scheme is stopped after the values of P and ϕ_w change by less than 0.1%. The values of $\rho_{\mathrm{L},i}$ and $\mu^\mathrm{ex}_{\mathrm{L},i}$ are for the liquid phase. Due to the incompressibility of the liquid phase, these are independent of changes in pressure (at least below 50 bar).[18] For the Peng-Robinson equation of state for water in the gas phase the following parameters are used: critical temperature of 647.3 K, critical pressure of 221.2 bar, and an acentric factor of 0.344.[25]

S3 Derivation of the Partition function and the Free Energy Correction

In this section, we derive an expression for the partition function in the isobaric-isothermal ensemble (Q_{NPT}) after applying the free energy correction to the isolated molecule partition function of water in the liquid phase (as discussed in the main text).

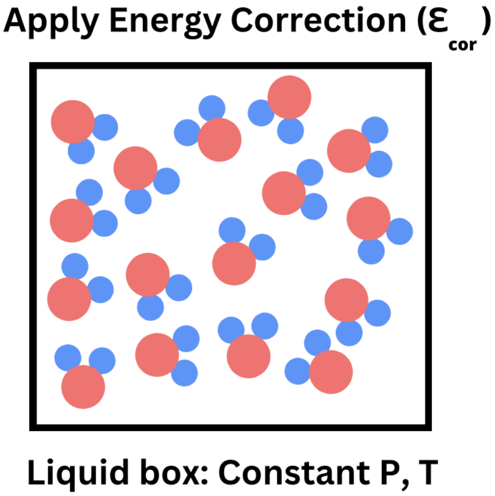

Figure S1: Simulation box of liquid water at constant temperature (T) and pressure (P). A background energy for component i (ϵ_i) is applied to modify the free energies computed from the Potential Energy Surface (PES).

A schematic representation of the simulation box is shown in Figure S1. In the simulation box (liquid phase), molecules are subjected to a background potential that adds an additional temperature-dependent energy contribution (ϵ_i) to the isolated molecule partition function (q_i) of molecule type i. We will derive how this background energy changes the partition function of the system. The partition function in the isobaric isothermal ensemble (Q_{NPT}) for a mixture of n_t components is equal to:[19,23]

$$Q_{\rm NPT} = \frac{P}{k_{\rm B}T}\left(\prod_{i=1}^{n_t}\frac{q_i^{N_i}}{N_i!\Lambda_i^{3N}}\right)\int {\rm d}V V^N \exp\left[\frac{-PV}{k_{\rm B}T}\right]\int {\rm d}\mathbf{s}^N \exp\left[\frac{-U(\mathbf{s}^N)}{k_{\rm B}T}\right] \tag{S8}$$

where P, V, $k_{\rm B}$, T, Λ_i , N, $\mathbf{s}^N$, and $U(\mathbf{s}^N)$ refer to pressure, volume, Boltzmann constant,

temperature, the thermal wavelength of component i, total number of molecules, the scaled position vector of all molecules, and the internal energy of the system (a function of $\mathbf{s}^N$). q_i and N_i are the isolated molecular partition functions (excluding the translational part) and the number of molecules of type i, respectively. q_i can be expressed as:[23]

$$q_i = \sum_j \exp\left[-\frac{\epsilon_{\mathrm{el},j} + \epsilon_{\mathrm{rot},j} + \epsilon_{\mathrm{vib},j} + \epsilon_i}{k_{\mathrm{B}}T}\right] \tag{S9}$$

$\epsilon_{\mathrm{el},j}$, $\epsilon_{\mathrm{rot},j}$, $\epsilon_{\mathrm{vib},j}$, are the electronic, rotational, and vibrational energy of intramolecular system state j. As these do not depend on the free energy correction ϵ_i, we can group these terms together in a term $q_{0,i}$. ϵ_i is not a function of the intramolecular system state j and can be factored out of the summation:

$$q_i = q_{0,i} \exp\left[\frac{\epsilon_i}{k_{\mathrm{B}}T}\right] \tag{S10}$$

Combining Eq. S10 and Eq. S8, we obtain:

$$Q_{\mathrm{NPT}} = \frac{P}{k_{\mathrm{B}}T}\left(\prod_{i=1}^{n_t} \frac{q_{0,i}^{N_i}\exp\left[\frac{N_i\epsilon_i}{k_BT}\right]}{N_i!\Lambda_i^{3N}}\right)\int \mathrm{d}V V^N \exp\left[\frac{-PV}{k_{\mathrm{B}}T}\right]\int \mathrm{d}\mathbf{s}^N \exp\left[\frac{-U(\mathbf{s}^N)}{k_{\mathrm{B}}T}\right] \tag{S11}$$

As multiplying exponential functions is equivalent to summing the exponents, we have:

$$\prod_{i=1}^{n_t}\exp\left[\frac{N_i\epsilon_i}{k_{\mathrm{B}}T}\right] = \exp\left[\sum_i^{n_t}\frac{N_i\epsilon_i}{k_{\mathrm{B}}T}\right] \tag{S12}$$

Note that ϵ_i (a function of T) is not a function of V or $\mathbf{s}^N$. Therefore, Eq. S11 can be reformulated as:

$$Q_{\mathrm{NPT}} = \frac{P}{k_{\mathrm{B}}T}\left(\prod_{i=1}^{n_t} \frac{q_{0,i}^{N_i}}{N_i!\Lambda_i^{3N}}\right)\int \mathrm{d}V V^N \exp\left[\frac{-PV}{k_{\mathrm{B}}T}\right]\int \mathrm{d}\mathbf{s}^N \exp\left[\frac{-U(\mathbf{s}^N) + \sum_i^{n_t} N_i\epsilon_i}{k_{\mathrm{B}}T}\right] \tag{S13}$$

The ensemble averaged volume ($\langle V \rangle$) can be computed by taking the partial derivative of $\ln(Q_{\text{NPT}})$ with respect to P at constant T and N (for all molecular species):[23]

$$\langle V \rangle = -k_{\text{B}}T \left(\frac{\partial \ln(Q_{NPT})}{\partial P} \right)_{T,N} \tag{S14}$$

The chemical potential of species i (μ_i) can be computed by taking the partial derivative of $\ln(Q_{\text{NPT}})$ with respect to N_i at constant T, P, and N_j (for $j \neq i$):

$$\mu_i = -k_{\text{B}}T \left(\frac{\partial \ln(Q_{NPT})}{\partial N_i} \right)_{T,P,N_{j,j\neq i}} \tag{S15}$$

For computing $\langle V \rangle$ and μ_i, we first need to evaluate $\ln(Q_{NPT})$. Considering that multiplication inside the natural logarithm is equal to the summation of the natural logarithms (i.e., $\ln(AB) = \ln(A) + \ln(B)$) and that ϵ_i is not a function of $\mathbf{s}^N$, we can express $\ln(Q_{NPT})$ as:

$$\begin{aligned} \ln(Q_{\text{NPT}}) = \ln &\left(\frac{P}{k_{\text{B}}T} \left(\prod_i^{n_t} \frac{q_{0,i}^{N_i}}{N_i! \Lambda_i^{3N}} \right) \int \text{d}V V^N \exp\left[\frac{-PV}{k_{\text{B}}T} \right] \int \text{d}\mathbf{s}^N \exp\left[\frac{-U(\mathbf{s}^N)}{k_{\text{B}}T} \right] \right) \\ &+ \sum_i^{n_t} \frac{N_i \epsilon_i}{k_{\text{B}}T} \end{aligned} \tag{S16}$$

Taking the partial derivative of $\ln(Q_{NPT})$ with respect to P results in cancellation of the terms containing ϵ_i, as these are not functions of P (and only a weak function of T, as explained in the main text). This entails that $\langle V \rangle$ does not depend on the correction term ϵ_i and hence the pressure of the system is unaltered. Evaluating Eq. S15 results in three seperate terms for the chemical potential of species i:

$$\mu_i = \mu_i^{\text{id}} + \mu_{i,\text{PES}}^{\text{ex}} + \epsilon_i \tag{S17}$$

The first term is the ideal gas term (μ_i^{id}), which depends on the isolated molecule partition function (excluding the contribution of ϵ_i) and the density. $\mu_{i,\text{PES}}^{\text{ex}}$ is determined by the force field used to describe the PES. The third term is the correction term, which shifts

the chemical potential of species i obtained using the PES by ϵ_i. This adjustment to the partition function ensures that the excess chemical potentials are corrected without changing $\langle V \rangle$, transport properties, the liquid structure, and the pressure.

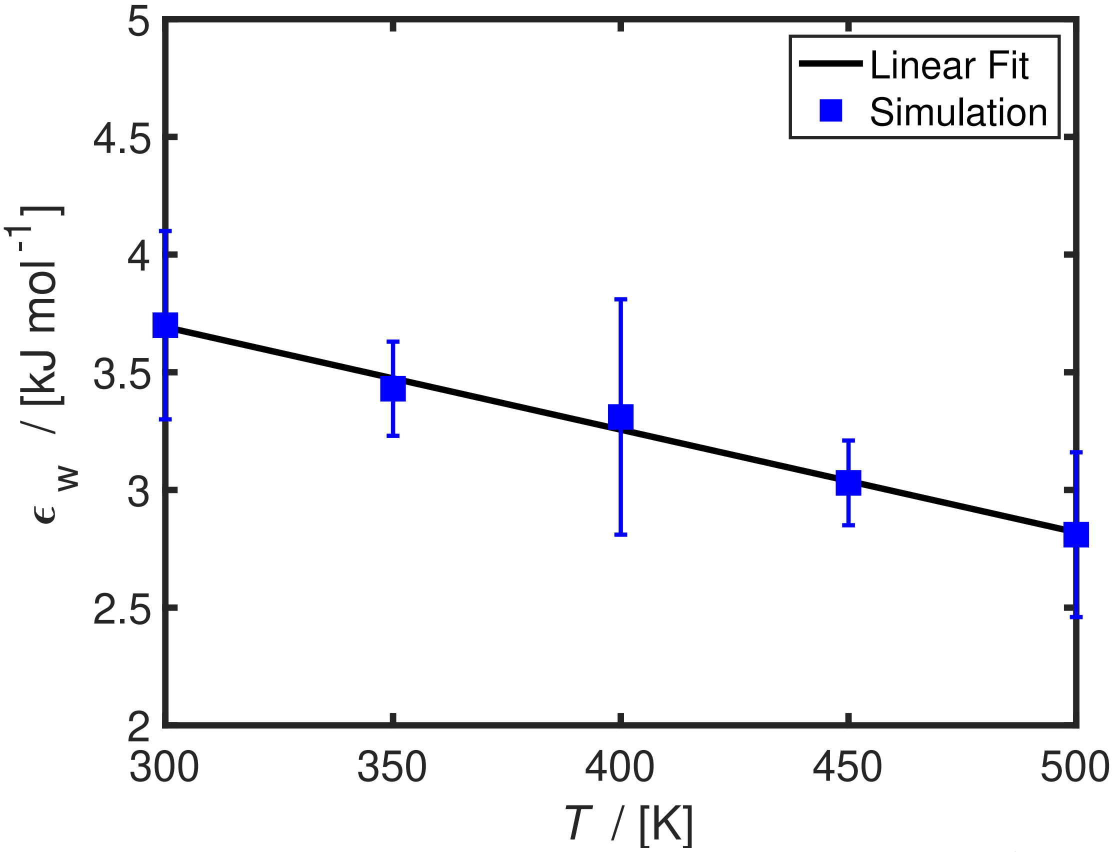

Figure S2: Computed free energy corrections of water (ϵ_{w} in units of kJ mol^{-1}) as a function of temperature (T in units of K). ϵ_{w} is defined as $\epsilon_{\mathrm{w}} = \mu^{\mathrm{ex}}_{\mathrm{s,ECS},m=0} - \mu^{\mathrm{ex}}_{\mathrm{s,PES},m=0}$, where $\mu^{\mathrm{ex}}_{\mathrm{s,ECS},m=0}$ and $\mu^{\mathrm{ex}}_{\mathrm{s,PES},m=0}$ refer to the excess chemical potentials (with respect to the ideal gas reference state) at a salt molality (m) of 0 computed using the Effective Charge Surface (ECS) and the TIP4P/2005[1] force field (PES), respectively. ϵ_{w} is fitted as a linear equation of T (Eq. 8 of the main text). The parameters of this fit can be found in the main text.

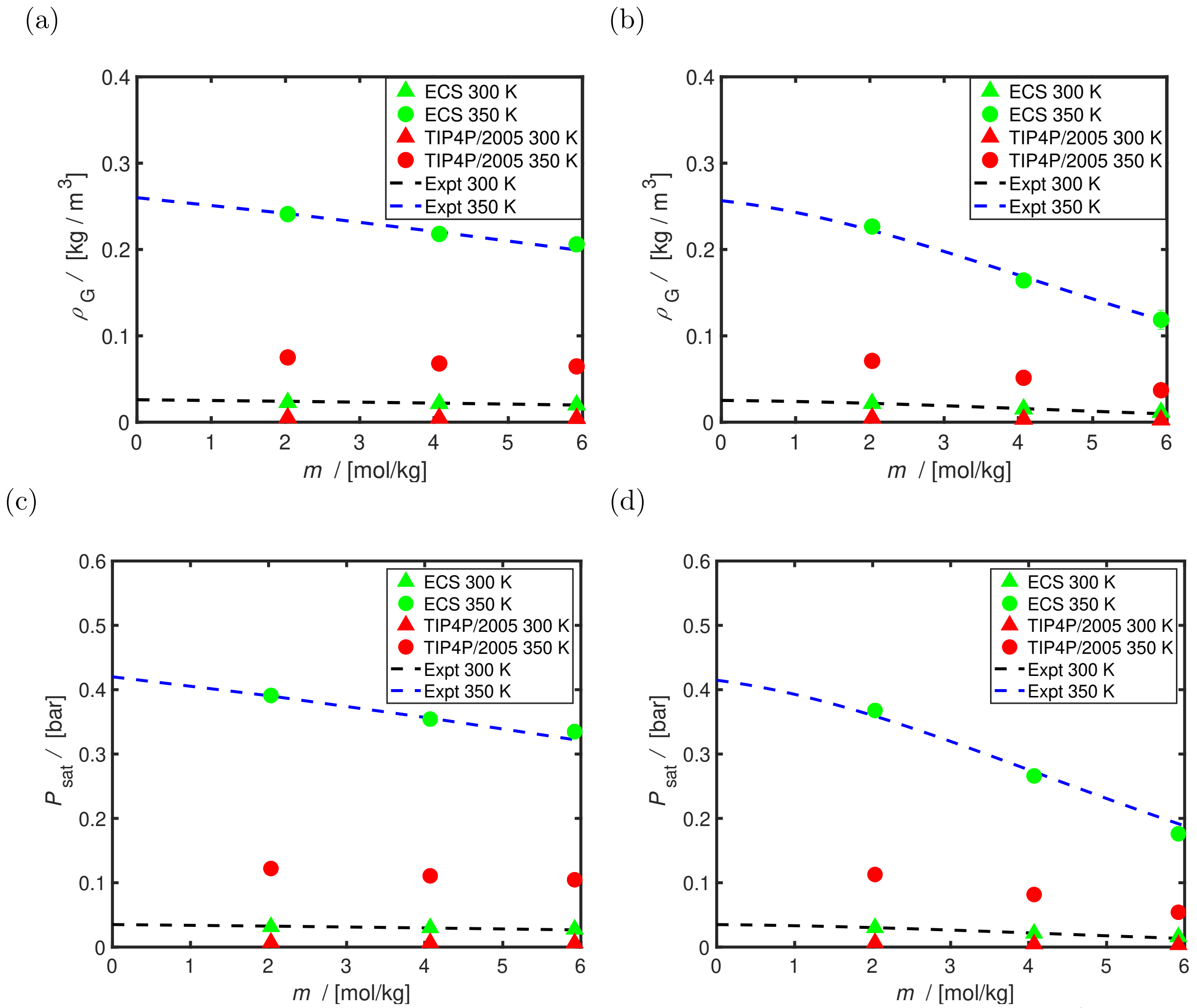

Figure S3: Computed saturated vapor densities of water (ρ_G) in units of kg m^{-3} for aqueous (a) NaCl and (b) $CaCl_2$ solutions and computed saturated vapor pressures (P_{sat}) in units of bar for aqueous (c) NaCl and (d) $CaCl_2$ solutions, at 300 K and 350 K. The ECS results (i.e., using the free energy correction as described in the main text) are compared to the results of TIP4P/2005 without the correction. The Madrid-2019[12] force fields of NaCl and $CaCl_2$ are used. The experimental data of Clarke and Glew[26] and Sako et al.[27] are used for aqueous NaCl and $CaCl_2$ solutions, respectively. The ECS results can accurately predict the experimental results. Without the free energy correction, the results of TIP4P/2005 combined with the Madrid-2019 force field deviate by a factor of ca. 4 from the experiments. The liquid densities and excess chemical potentials used to calculate ρ_G and P_{sat} from Eq. 5 of the main text are computed at 1 bar. All the raw data can be found in Table S5.

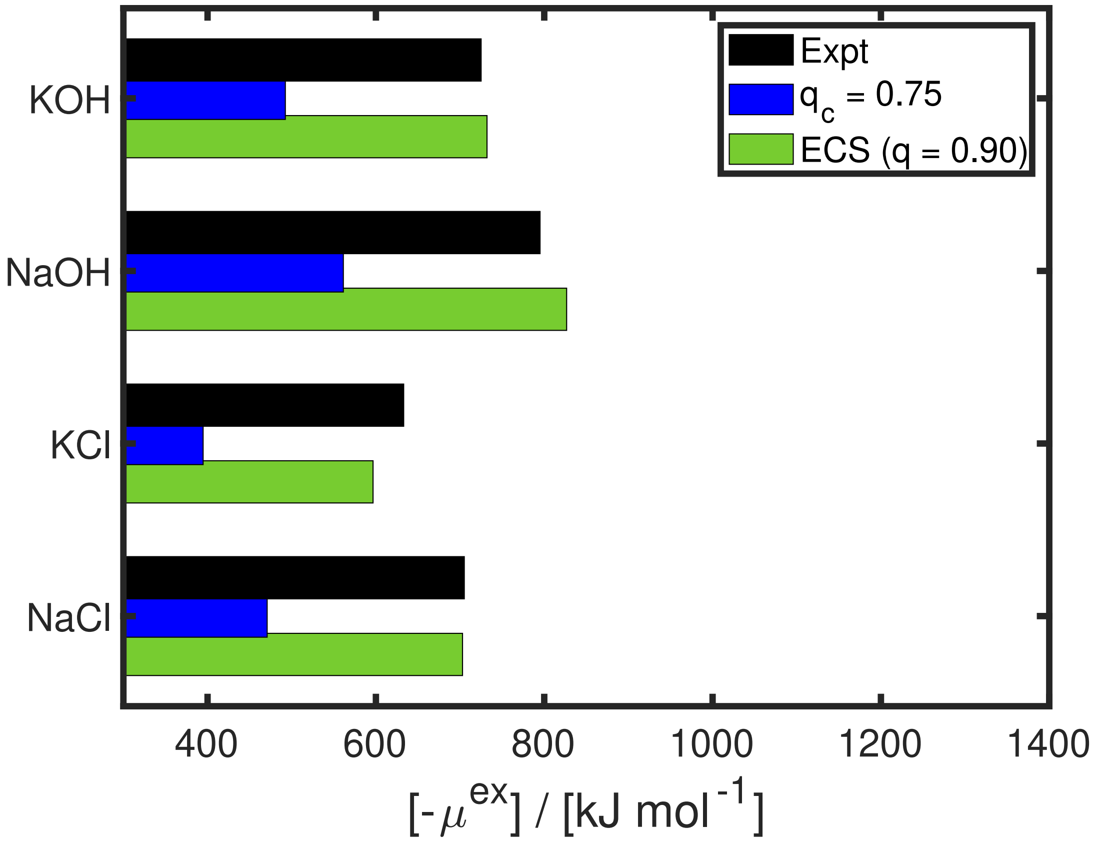

Figure S4: Computed infinite dilution excess chemical potentials (μ_{ex}) (i.e., free energies of hydration, with respect to ideal gas reference state) at 298 K and 1 bar for aqueous NaCl, KCl, NaOH, and KOH solutions at infinite dilution. The ion force fields of Madrid-Transport[13] and Delft Force Field of OH^-(DFF/OH^-) (scaled charges of +0.75/-0.75) are used. The TIP4P/2005[1] water force field is used for all calculations. In the ECS approach, a single fractional group of cations and anions are used with the same LJ parameters of the Madrid-Transport[13] and DFF/OH^- force fields. Ion charges of +0.90/-0.90 for monovalent ions are used to sample the free energies of hydration (fitted only to the free energy of hydration of an aqueous NaCl solution at infinite dilution). The experimental results of Marcus[16] are shown in black. Different ECS charges of +0.95/-0.95 are used for the Madrid-2019[12] ion force fields with scaled charges of +0.85/-0.85. This different ECS can be attributed to having differing Lennard-Jones parameters depending on the scaled charges (i.e., 0.75[13] and 0.85[12]). The raw data are shown in Table S7 of the Supporting Information.

Literature Cited

(1) Abascal, J. L.; Vega, C. A general purpose model for the condensed phases of water: TIP4P/2005. *J. Chem. Phys.* **2005**, *123*, 234505.

(2) Rahbari, A.; Hens, R.; Ramdin, M.; Moultos, O. A.; Dubbeldam, D.; Vlugt, T. J. H. Recent Advances in the Continuous Fractional Component Monte Carlo Methodology. *Mol. Simulat.* **2021**, *47*, 804–823.

(3) Rahbari, A.; Garcia-Navarro, J. C.; Ramdin, M.; van den Broeke, L. J. P.; Moultos, O. A.; Dubbeldam, D.; Vlugt, T. J. H. Effect of Water Content on Thermodynamic Properties of Compressed Hydrogen. *J. Chem. amp; Eng. Data* **2021**, *66*, 2071–2087.

(4) Shi, W.; Maginn, E. J. Continuous Fractional Component Monte Carlo: an Adaptive Biasing Method for Open System Atomistic Simulations. *J. Chem. Theory Comput.* **2007**, *3*, 1451–1463.

(5) Shi, W.; Maginn, E. J. Improvement in Molecule Exchange Efficiency in Gibbs Ensemble Monte Carlo: Development and Implementation of the Continuous Fractional Component Move. *J. Comput. Chem.* **2008**, *29*, 2520–2530.

(6) Hens, R.; Rahbari, A.; Caro-Ortiz, S.; Dawass, N.; Erdős, M.; Poursaeidesfahani, A.; Salehi, H. S.; Celebi, A. T.; Ramdin, M.; Moultos, O. A.; Dubbeldam, D.; Vlugt, T. J. H. Brick-CFCMC: Open Source Software for Monte Carlo Simulations of Phase and Reaction Equilibria Using the Continuous Fractional Component Method. *J. Chem. Inf. Model.* **2020**, *60*, 2678–2682.

(7) Polat, H. M.; Salehi, H. S.; Hens, R.; Wasik, D. O.; Rahbari, A.; de Meyer, F.; Houriez, C.; Coquelet, C.; Calero, S.; Dubbeldam, D.; Moultos, O. A.; Vlugt, T. J. H. New Features of the Open Source Monte Carlo Software Brick-CFCMC: Thermodynamic Integration and Hybrid Trial Moves. *J. Chem. Inf. Model.* **2021**, *61*, 3752–3757.

(8) Wagner, W.; Pruß, A. The IAPWS Formulation 1995 for the Thermodynamic Properties of Ordinary Water Substance for General and Scientific Use. *J. Phys. Chem. Ref. Data* **2002**, *31*, 387–535.

(9) Lemmon, E. W.; Bell, I. H.; Huber, M. L.; McLinden, M. O. NIST Standard Reference Database 23: Reference Fluid Thermodynamic and Transport Properties-REFPROP, Version 10.0, National Institute of Standards and Technology. 2018; `https://www.nist.gov/srd/refprop`.

(10) Lopez-Echeverry, J. S.; Reif-Acherman, S.; Araujo-Lopez, E. Peng-Robinson Equation of State: 40 years Through Cubics. *Fluid Ph. Equilib.* **2017**, *447*, 39–71.

(11) Peng, D.-Y.; Robinson, D. B. A New Two-Constant Equation of State. *Ind. Eng. Chem. Fund.* **1976**, *15*, 59–64.

(12) Zeron, I. M.; Abascal, J. L. F.; Vega, C. A Force Field of Li^+, Na^+, K^+, Mg^{2+}, Ca^+, Cl^-, and SO_4^{2-} in Aqueous Solution Based on the TIP4P/2005 Water Model and Scaled Charges for the Ions. *J. Chem. Phys.* **2019**, *151*, 104501.

(13) Blazquez, S.; Conde, M. M.; Vega, C. A. Scaled Charges for Ions: An Improvement but not the Final Word for Modeling Electrolytes in Water. *J. Chem. Phys.* **2023**, *158*, 054505.

(14) Joung, I. S.; Cheatham, T. E. I. Determination of Alkali and Halide Monovalent Ion Parameters for Use in Explicitly Solvated Biomolecular Simulations. *J. Phys. Chem. B* **2008**, *112*, 9020–9041.

(15) Benavides, A. L.; Aragones, J. L.; Vega, C. Consensus on the Solubility of NaCl in Water from Computer Simulations Using the Chemical Potential Route. *J. Chem. Phys.* **2016**, *144*, 124504.

(16) Marcus, Y. A Simple Empirical Model Describing the Thermodynamics of Hydration of Ions of Widely Varying Charges, Sizes, and Shapes. *Biophys. Chem.* **1994**, *51*, 111–127.

(17) Schmid, R.; Miah, A. M.; Sapunov, V. N. A New Table of the Thermodynamic Quantities of Ionic Hydration: Values and Some Applications (Enthalpy–Entropy Compensation and Born Radii). *Phys. Chem. Chem. Phys.* **2000**, *2*, 97–102.

(18) Habibi, P.; Rahbari, A.; Blazquez, S.; Vega, C.; Dey, P.; Vlugt, T. J. H.; Moultos, O. A. A New Force Field for OH^- for Computing Thermodynamic and Transport Properties of H_2 and O_2 in Aqueous NaOH and KOH Solutions. *J. Phys. Chem. B* **2022**, *126*, 9376–9387.

(19) Frenkel, D.; Smit, B. *Understanding Molecular Simulation: from Algorithms to Applications*, 3rd ed.; Elsevier: San Diego, 2023.

(20) Wang, F.; Landau, D. P. Efficient multiple-range random walk algorithm to calculate the density of states. *Phys. Rev. Letters* **2001**, *86*, 2050.

(21) Poulain, P.; Calvo, F.; Antoine, R.; Broyer, M.; Dugourd, P. Performances of Wang-Landau algorithms for continuous systems. *Phys. Rev. E* **2006**, *73*, 056704.

(22) van Rooijen, W. A.; Habibi, P.; Xu, K.; Dey, P.; Vlugt, T. J. H.; Hajibeygi, H.; Moultos, O. A. Interfacial Tensions, Solubilities, and Transport Properties of the H2/H2O/NaCl System: A Molecular Simulation Study. *J. Chem. amp; Eng. Data* **2024**, *69*, 307–319.

(23) Sandler, S. I. *An Introduction to Applied Statistical Thermodynamics*, 1st ed.; John Wiley & Sons: Hoboken, New Jersey, 2011.

(24) Rahbari, A.; Brenkman, J.; Hens, R.; Ramdin, M.; Broeke, L. J. V. D.; Schoon, R.; Henkes, R.; Moultos, O. A.; Vlugt, T. J. H. Solubility of water in hydrogen at high

pressures: A molecular simulation study. *J. Chem. amp; Eng. Data* **2019**, *64*, 4103–4115.

(25) R. C. Reid, J. M. P.; Poling, B. E. *The Properties of Gases and Liquids*, 5th ed.; McGraw-Hill: New York, 2001.

(26) Clarke, E. C. W.; Glew, D. N. Evaluation of the Thermodynamic Functions for Aqueous Sodium Chloride from Equilibrium and Calorimetric Measurements below 154°C. *J. Phys. Chem. Ref. Data* **1985**, *14*, 489–610.

(27) Sako, T.; Hakuta, T.; Yoshitome, H. Vapor Pressures of Binary (Water-Hydrogen Chloride, -Magnesium Chloride, and -Calcium Chloride) and Ternary (Water-Magnesium Chloride-Calcium Chloride) Aqueous Solutions. *J. Chem. amp; Eng. Data* **1985**, *30*, 224–228.
