# Supplementary material for: Accurate Free Energies of Aqueous Electrolyte Solutions from Molecular Simulations with Non-polarizable Force Fields
Source: J Phys Chem Lett. 2024 Apr 18;15(16):4477–85. doi: 10.1021/acs.jpclett.4c00428 (PMC11057036; doi:10.1021/acs.jpclett.4c00428)
Supplement: Supplementary file 2 — jz4c00428_si_002.pdf [file jz4c00428_si_002.pdf]

jz-2024-00428h.R1

Name: Peer Review Information for "Accurate Free Energies of Aqueous Electrolyte Solutions from Molecular Simulations with Non-Polarizable Force Fields"

First Round of Reviewer Comments

Reviewer: 1

Comments to the Author

Journal of Physical Chemistry Letters Review of jz-2024-00428h

Accurate Free Energies of Aqueous Electrolyte Solutions from Molecular Simulations with Non-Polarizable Force Fields

This paper proposes a novel ECS methodology for adjusting the excess chemical potential, μ^{ex}, of the liquid TIP4P/2005 non-polarizable water force field, for the purpose of improving its liquid phase chemical potential for calculating the vapour pressure and coexisting phase densities of aqueous electrolyte solutions, while at the same time keeping intact its predictive abilities for density and transport properties.

Fig. 2 makes the case that the problem with the TIP4P/2005 force field (FF) is its poor liquid phase μ^{ex} predictions for pure water, which result in poor predictions of P_{sat} and its coexistence vapor density. Figs. 3(a) and 3(b) then demonstrate that for electrolyte solutions, TIP4P/2005 gives good predictions of the water activity, and hence the problem for aqueous electrolyte VLE prediction can be traced back to the problem with the μ^{ex} component of its pure component chemical potential $\mu_w(T,P)$.

To repair the TIP4P/2005 liquid phase μ^{ex} deficiencies, the authors adjusted it by fitting an "ECS correction" for the water chemical potential at infinite dilution to the experimental μ^{ex} value at 350 K, which (quoting from the Abstract) "enables accurate predictions of Vapor-Liquid Equilibria of aqueous electrolytes using a single non-polarizable force field, without compromising the liquid phase properties."

As a related application, the authors describe the use of their ECS approach to correct the infinite dilution hydration free energies of several aqueous ions to the experimental values by adjusting their force-field partial charges. This is essentially a "mirror image" of the same

procedure they used to adjust the pure water chemical potential value for the purpose of calculating the vapor pressure..

The paper will be of interest to researchers working in the field of atomistic molecular simulations of aqueous electrolyte mixtures, but it requires revision as indicated below before it is acceptable.

Comments:

1. On p. 9 line 54, when referring to Eq. (5), the authors state that they used the PengRobinson equation of state to obtain the water vapor-phase fugacity coefficient ϕ. This is inconsistent with the use of a force field for the liquid phase but an experimentally based equation of state for the vapor phase. The authors should explain why they did not use the TIP4P/2005 force field to calculate ϕ for the vapor phase.

2. In accordance with the final sentence of the Abstract, the authors should add a sub-figure (d) to Fig. 3 that shows the dependence of the NaCl vapor pressure as a function of composition for TIP4P/2005 and ECS, in comparison with experiment.

3. The authors should add an additional figure that shows the dependence of the vapor pressureas a function of composition at several temperatures in comparison with experiment when the ECS is used for pure water. I suggest two sub-figures, of which (a) would be for NaCl and (b) would be for $CaCl_2$.

4. The provision of numerical data is crucially important for the research community. Thenumerical data for the current Figs. 2 and 3 and for the additional figure suggested in the previous point should be added to the SI.

5. On p. 9 near line 31, the word “saturated” should (presumably) be added before “liquid”.The authors quote a -26.2 kJ/mol for the experimental value of the saturation excess chemical potential obtained from REFPROP at 300K. However, Table S3 shows a value of -26.37 kJ/mol. This discrepancy should be remedied. Reference 53 for REFPROP quotes a very old version of REFPROP, which may be the source of the -26.2 value..

Reviewer: 2

Comments to the Author

The manuscript describes a method that uses non-polarizable force field calculations and that allows “accurately predict free-energies of aqueous electrolytes without compromising the predictive ability for density and transport properties.” The results do look impressive, although I am not sure I correctly understood the procedure used. Although computationally efficient, it seems to me that the method uses TIP4P/2005 charges for calculations of water properties such as viscosity, diffusivity, density, etc., while another set of charges, ECS charges, are used to calculate excess chemical potentials. i.e. two different potential surfaces are used to describe properties of physical system. This contradicts the statements made in the paper, e.g. the statement from the Abstract: “This approach enables accurate prediction of Liquid-Vapor Equilibria of aqueous electrolytes using a single non-polarizable force field, without compromising the liquid phase properties.” If indeed the properties of aqueous solutions need to be accurately described by two different sets of charges (even if the simulations are performed once), I think the authors should make it clear in the paper.

Author's Response to Peer Review Comments:

Manuscript ID: jz-2024-00428h

22 March 2024

Dear editor,

Many thanks for sending the reports of the reviewers for our manuscript titled "Accurate Free Energies of Aqueous Electrolyte Solutions from Molecular Simulations with Non-Polarizable Force Fields" by Parsa Habibi, H. Mert Polat, Samuel Blazquez, Carlos Vega, Poulumi Dey, Thijs J. H. Vlugt, and OthonasA. Moultos. We would like to thank the reviewers for their constructive comments. The manuscript is now revised based on their comments, and the formatting comments of the editor. A marked copy is uploaded with all changes indicated in red.

With the changes we implemented and given the positive reviews, we hope that the revised paper can be now accepted for publication in the journal of *Physical Chemistry Letters.*

Best regards,

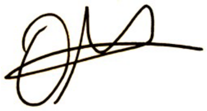

Othonas A. Moultos (on behalf of all the authors)

We would like to thank the reviewers for their comments. In this rebuttal, we first replicate the reviewers' points, and then present our responses in *italics*. Our revisions addressing the comments of the reviewers are shown in red in the marked version of the revised manuscript and Supporting Information document. The text in red here is excerpts from the revised manuscript and the revised Supporting Information.

Reviewer 1

This paper proposes a novel ECS methodology for adjusting the excess chemical potential, μ_ex, of the liquid TIP4P/2005 non-polarizable water force field, for the purpose of improving its liquid phase chemical potential for calculating the vapour pressure and coexisting phase densities of aqueous electrolyte solutions, while at the same time keeping intact its predictive abilities for density and transport properties. Fig. 2 makes the case that the problem with the TIP4P/2005 force field (FF) is its poor liquid phase μ_ex predictions for pure water, which result in poor predictions of P_sat and its coexistence vapor density. Figs. 3(a) and 3(b) then demonstrate that for electrolyte solutions, TIP4P/2005 gives good predictions of the water activity, and hence the problem for aqueous electrolyte VLE prediction can be traced back to the problem with the μ_ex component of its pure component chemical potential μw(T, P). To repair the TIP4P/2005 liquid phase μ_ex deficiencies, the authors adjusted it by fitting an "ECS correction" for the water chemical potential at infinite dilution to the experimental μ_ex value at 350 K, which (quoting from the Abstract) "enables accurate predictions of Vapor-Liquid Equilibria of aqueous electrolytes using a single non-polarizable force field, without compromising the liquid phase properties." As a related application, the authors describe the use of their ECS approach to correct the infinite dilution hydration free energies of several aqueous ions to the experimental values by adjusting their forcefield partial charges. This is essentially a "mirror image" of the same procedure they used to adjust the pure water chemical potential value for the purpose of calculating the vapor pressure. The paper will be of interest to researchers working in the field of atomistic molecular simulations of aqueous electrolyte mixtures, but it requires revision as indicated below before it is acceptable.

Response: *We thank the reviewer for his/her feedback and for recommending publication.*

1. On p. 9 line 54, when referring to Eq. (5), the authors state that they used the Peng-Robinson equation of state to obtain the water vapor-phase fugacity coefficient ϕ. This is inconsistent with the use of a force field for the liquid phase but an experimentally based equation of state for the vapor phase. The authors should explain why they did not use the TIP4P/2005 force field to calculate ϕ for the vapor phase.

Response: *As suggested by the reviewer, we have revised the manuscript to motivate the choice of using the Peng-Robinson equation of state to describe the gas phase instead of the TIP4P/2005 force field.*

Revision in manuscript (pages 9-10):

The TIP4P/2005 force field cannot accurately model the virial coefficient of water in the gas phase [34, 35], and hence, it does not correctly describe the deviations from ideal gas behavior, leading to an inaccurate relation between T, P_{sat}, and $\phi_{!}$.

2. In accordance with the final sentence of the Abstract, the authors should add a sub-figure (d) to Fig. 3 that shows the dependence of the NaCl vapor pressure as a function of composition for TIP4P/2005 and ECS, in comparison with experiment.

Response: *Experimental measurements show that NaCl is non-volatile (i.e., partial pressure is almost zero) at the temperatures, pressures, and compositions considered. This agrees with the highly negative free energy of hydration of NaCl obtained both in experiments and in our work. To calculate NaCl vapor pressures from simulations, the excess chemical potential of NaCl in the gas phase needs to be computed at multiple simulation volumes and extrapolated to infinite volume to correct for the effect of long-range Ewald electrostatic energies between molecules and their periodic images (as explained by Ref. 64 and 65 of the revised manuscript). We are convinced that these calculations are beyond the scope of this work. The manuscript is revised in to explain how NaCl pressures can be related to the free energies of hydration. Also, the final sentence of the abstract is revised to clarify the focus of this work which is on liquid phase free energies.*

Revision in manuscript (page 2):

This approach enables accurate predictions of free energies of aqueous electrolyte solutions using non-polarizable force fields, without compromising liquid phase properties.

Revision in manuscript (page 14-15):

The partial pressures of salts (e.g., NaCl) in the vapor phase (which is practically negligible) can be computed from the free energy of hydration and excess chemical potential of NaCl in the vapor phase (as discussed in Refs. [64, 65]), however, this is beyond the scope of this work.

3. The authors should add an additional figure that shows the dependence of the vapor pressure as a function of composition at several temperatures in comparison with experiment when the ECS is used for pure water. I suggest two sub-figures, of which (a) would be for NaCl and (b) would be for $CaCl_2$.

Response: *As suggested by the reviewer we have added a new figure (Figure S3 in the Supporting Information), which shows the variation of vapor densities as a function of composition at two different temperatures (300 K and 350 K) for two different salts (NaCl and* $CaCl_2$*). We have also revised the manuscript on page 13 as follows:*

The saturated vapor densities of aqueous NaCl and $CaCl_2$ *solutions are computed at 300-350 K and shown in Figure S3 of the Supporting Information. As shown in this figure, the ECS approach combined with TIP4P/2005 and the Madrid-2019 force fields can accurately capture the experimental saturated vapor densities for aqueous NaCl and* $CaCl_2$ *solutions (within 5% deviations).*

Revision in the Supporting Information (page S19):

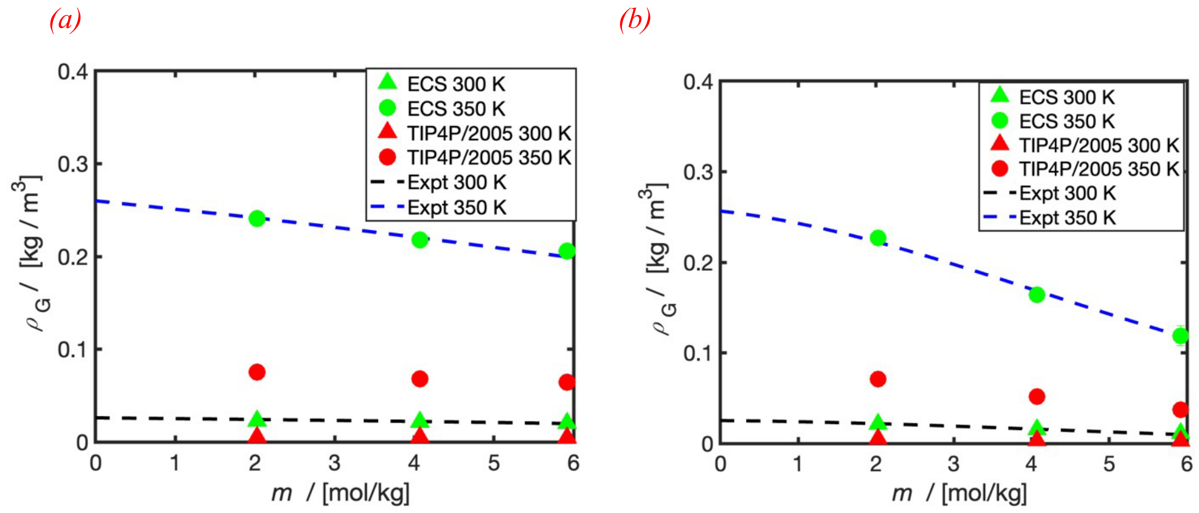

Figure S3. Computed saturated vapor densities (ρ''*) in units of kg* m^{-3} *for aqueous (a) NaCl and (b)* $CaCl_2$ *solutions, at 300 K and 350 K. The ECS results (i.e., using the free energy correction as described in the main text) are compared to the results of TIP4P/2005 without the correction. The Madrid-2019 [12] force fields of NaCl and* $CaCl_2$ *are used. The experimental data of Clarke and Glew [26] and Sako et al. [27] are used for aqueous NaCl and* $CaCl_2$ *solutions, respectively. The ECS results can accurately predict the experimental results. Without the free energy correction, the results of TIP4P/2005 combined with the Madrid-2019 force field deviate by a factor of ca. 4 from the experiments. All the raw data can be found in Table S5.*

4. The provision of numerical data is crucially important for the research community. The numerical data for the current Figs. 2 and 3 and for the additional figure suggested in the previous point should be added to the SI.

Response: *We agree with the reviewer. All numerical data produced in this work (including the additional figure from the previous point) are now provided in the Supporting Information (Tables S3-S7 of the revised version).*

5. On p. 9 near line 31, the word “saturated” should (presumably) be added before “liquid”. The authors quote a -26.2 kJ/mol for the experimental value of the saturation excess chemical potential obtained from REFPROP at 300K. However, Table S3 shows a value of -26.37 kJ/mol. This discrepancy should be remedied. Reference 53 for REFPROP quotes a very old version of REFPROP, which may be the source of the -26.2 value.

Response: *We thank the reviewer for spotting this inconsistency. The cited version of REFPROP is now updated (version 10). The value of -26.37 kJ/mol is the correct value based on IAPWS-95 (Ref. 55 of the revised manuscript). The manuscript has been corrected on page 9 as follows:*

....the experimental value is -26.37 kJ/mol (REFPROP version 10 [54], computed based on IAPWS-95 [55]).

Reviewer 2

The manuscript describes a method that uses non-polarizable force field calculations and that allows “accurately predict free-energies of aqueous electrolytes without compromising the predictive ability for density and transport properties.” The results do look impressive, although I am not sure I correctly understood the procedure used. Although computationally efficient, it seems to me that the method uses TIP4P/2005 charges for calculations of water properties such as viscosity, diffusivity, density, etc., while another set of charges, ECS charges, are used to calculate excess chemical potentials. i.e. two different potential surfaces are used to describe properties of physical system. This contradicts the statements made in the paper, e.g. the statement from the Abstract: “This approach enables accurate prediction of Liquid-Vapor Equilibria of aqueous electrolytes using a single non-polarizable force field, without compromising the liquid phase properties.” If indeed the properties of aqueous solutions need to be accurately described by two different sets of charges (even if the simulations are performed once), I think the authors should make it clear in the paper.

Response: *We thank the reviewer for recommending publication. We understand that the last sentence of the abstract may be misleading, and it is therefore revised (see the answer to comment 1 by reviewer 1).*

To clarify that there are two charge surfaces (one for liquid water properties and another for computing free energies) an additional sentence is added to the conclusions part on page 15 of the revised manuscript:

In summary, we have shown that non-polarizable rigid force fields (here we use TIP4P/2005) can accurately model the vapor-liquid properties of aqueous electrolyte solutions, provided that an additional charge surface, the so-called Effective Charge Surface (ECS), is used to correct the infinite dilution excess chemical potentials of water and salts, while TIP4P/2005 and scaled ion charges are used to compute densities, activities, and transport properties of the liquid phase.

jz-2024-00428h.R2

Name: Peer Review Information for "Accurate Free Energies of Aqueous Electrolyte Solutions from Molecular Simulations with Non-Polarizable Force Fields"

Second Round of Reviewer Comments

Reviewer: 1

Comments to the Author

Journal of Physical Chemistry Letters Review of Revision R1 jz-2024-00428hR1

Accurate Free Energies of Aqueous Electrolyte Solutions from Molecular Simulations with Non-Polarizable Force Fields

Further revisions are required before I would consider the manuscript to be acceptable for publication. This is mainly due to their misinterpretation of my previous Comments #2 and #3, to which I refer in the first 2 points below. Three additional points have been added.

Comments:

1. My previous Comment 2 may not have been sufficiently clear, but it refers to the vapourpressures of the aqueous NaCl solutions, whereas in their response the authors have taken it to refer to calculations of the NaCl partial pressures in the vapour. I agree with the authors that such a calculataion is outside the scope of this paper. The authors should remove their suggested revision on pp. 14-15..

2. Comment 3 of my previous review also is meant to refer to the *solution vapour pressure*. However, in their response, they have added a new Figure S3 to the Supporting Information that shows the

dependence of the saturated *vapour densities* of aqueous NaCl and $CaCl_2$. This figure is very useful information; however, I believe that the caption should refer to Table S5. In addition, it makes Fig. 3(c) of the main text redundant.

The authors should respond to my Comment concerning figures for the vapour pressures, which I have clarified and repeated below.

In accordance with the final sentence of the Abstract, the authors should add a sub-figure (d) to Fig. 3 that shows the dependence of the aqueous NaCl solution vapor pressure as a function of composition for TIP4P/2005 and ECS, and their comparison with experiment.

As a suggestion, the figure could alternatively be in the SI following the current Fig. S3, and the vapour pressure data could be included as an extra column in Table S5.

3. The reference for Eq. (5) of the main text is unclear. It was indicated as [33] in the maintext and [24] in the SI (see Eq. (S6)). However, I can't find that equation anywhere in either reference. In any event Eq. (5) is only valid for an incompressible fluid.

4. The authors have apparently assumed in their calculations that the liquid solutions areincompressible. This should be stated wherever appropriate, and the pressure (presumably 1 bar?) actually used in the simulations should be indicated in the appropriate places (see also Point #3).

5. Table S4 would be clearer if in the first row "Model" were replaced by "NaCl Model"

1

Author's Response to Peer Review Comments:

Manuscript ID: jz-2024-00428h.R1

Dear editor,

Many thanks for sending the second round of comments for our manuscript titled "Accurate Free

Energies of Aqueous Electrolyte Solutions from Molecular Simulations with Non-Polarizable Force Fields" by Parsa Habibi, H. Mert Polat, Samuel Blazquez, Carlos Vega, Poulumi Dey, Thijs J. H. Vlugt,

and OthonasA. Moultos. We would like to thank the reviewer for her/his constructive comments. The manuscript is now revised based on these comments. A marked copy is uploaded with all changes indicated in red.

With the changes that we have implemented, we hope that the revised paper can be now accepted for publication in the journal of *Physical Chemistry Letters.*

Best regards,

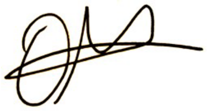

Othonas A. Moultos (on behalf of all the authors)

In this rebuttal, we first replicate the reviewer's comments and then present our responses in *italics*. Our revisions in the manuscript addressing the comments of the reviewer are shown in red. The text in red here are excerpts from the revised manuscript and the Supporting Information.

Response to the comments of Reviewer 1

Further revisions are required before I would consider the manuscript to be acceptable for publication. This is mainly due to their misinterpretation of my previous Comments #2 and #3, to which I refer in the first 2 points below. Three additional points have been added.

1. My previous Comment 2 may not have been sufficiently clear, but it refers to the vapour pressures of the aqueous NaCl solutions, whereas in their response the authors have taken it to refer to calculations of the NaCl partial pressures in the vapour. I agree with the authors that such a calculataion is outside the scope of this paper. The authors should remove their suggested revision on pp. 14-15.

Response: *We had indeed misinterpreted "NaCl vapour pressure" as "NaCl partial pressures". We have removed the revision on pp. 14-15 as suggested by the reviewer.*

2. Comment 3 of my previous review also is meant to refer to the solution vapour pressure. However, in their response, they have added a new Figure S3 to the Supporting Information that shows the dependence of the saturated vapour densities of aqueous NaCl and CaCl2. This figure is very useful information; however, I believe that the caption should refer to Table S5. In addition, it makes Fig. 3(c) of the main text redundant. The authors should respond to my Comment concerning figures for the vapour pressures, which I have clarified and repeated below.
In accordance with the final sentence of the Abstract, the authors should add a sub-figure (d) to Fig. 3 that shows the dependence of the aqueous NaCl solution vapor pressure as a function of composition for TIP4P/2005 and ECS, and their comparison with experiment. As a suggestion, the figure could alternatively be in the SI following the current Fig. S3, and the vapour pressure data could be included as an extra column in Table S5.

Response: *As pointed out by the reviewer, the caption of Figure S3 refers to Table S5. This typo is now corrected in the revised SI. We have added a sub-figure (d) to Figure 3 (on page 12 of the revised manuscript), which shows saturated vapor pressure as function of NaCl molalities. We have also added sub-figures (c) and (d) in Figure S3 of the Supporting Information (page S20), which show the saturated vapor pressures of aqueous NaCl and $CaCl_2$ solutions. In Table S5, two additional columns are added (for the average saturated vapor pressure data and the corresponding standard deviations).*

Revision in manuscript (page 13):

As shown in Figure 3(c) and 3(d), the ECS approach results in perfect agreement (within the error bars) with the data of Clarke and Glew [61] for the vapor phase coexistence pressures and densities of water/NaCl mixtures,…

2

3. The reference for Eq. (5) of the main text is unclear. It was indicated as [33] in the main text and [24] in the SI (see Eq. (S6)). However, I can't find that equation anywhere in either reference. In any event Eq. (5) is only valid for an incompressible fluid.

Response: *Eq (5) and the iterative scheme described in Section S2 of the Supporting Information is indeed valid for an incompressible liquid phase. The manuscript is revised in page 9 to clarify this aspect. Eq. (5) (for an ideal gas phase) is derived in Ref. [33] of the revised manuscript (page S9 of the Supporting Information of Ref. [33]). We have extended this equation to a non-ideal gas phase using the fugacity expression of Ref. [22] of the SI (on page S3 of the Supporting Information of Ref. [22]). Eq. (5) is derived in Section S2 of the Supporting Information of this manuscript, as indicated on page 9 of the manuscript.*

Revision in manuscript (page 10):

When calculating P!"# using Eq. 5, it is assumed that the liquid phase is incompressible (i.e., ρ$ and μ%&' computed in the liquid phase are not influenced by pressure in the range of 1-50 bar).

4. The authors have apparently assumed in their calculations that the liquid solutions are incompressible. This should be stated wherever appropriate, and the pressure (presumably 1 bar?) actually used in the simulations should be indicated in the appropriate places (see also Point #3).

Response: *We thank the reviewer for this suggestion. We have revised the manuscript to include the pressure used (in the simulations) to calculate the liquid phase densities and excess chemical potentials (in caption of Figure 3, Figure S3, and Table S5).*

Revision in manuscript (Figure 3 caption, page 13):

The liquid densities and excess chemical potentials used to calculate ρ(and P!"# from Eq. 5 are computed at 1 bar.

Revision in Supporting Information (Table S5 caption, page S6):

ρ$ and μ&',$ used to calculate ρ(and P!"# from Eq. 5 of the main text are computed at 1 bar.

Revision in Supporting Information (Figure S3 caption, page 20):

The liquid densities and excess chemical potentials used to calculate ρ(and P!"# from Eq. 5 of the main text are computed at 1 bar.

5. Table S4 would be clearer if in the first row "Model" were replaced by "NaCl Model"

Response: *As suggested by the reviewer the first row of Table S4 is now changed to "NaCl Model".*

3
